# Ling-gui-zhu-gan granules reduces obesity and ameliorates metabolic disorders by inducing white adipose tissue browning in obese mice

**DOI:** 10.3389/fphys.2024.1427722

**Published:** 2024-08-02

**Authors:** Yuxiu Li, Zimengwei Ye, Yi Zhao, Bingrui Xu, Wanying Xue, Zhufeng Wang, Ran An, Fan Wang, Rui Wu

**Affiliations:** ^1^ Department of Endocrinology, Guang’anmen Hospital South Campus, China Academy of Chinese Medical Sciences, Beijing, China; ^2^ School of Traditional Chinese Medicine, Beijing University of Chinese Medicine, Beijing, China; ^3^ College of Integrative Chinese and Western Medicine, Anhui University of Chinese Medicine, Hefei, China; ^4^ Department of Endocrinology, Guang’anmen Hospital, China Academy of Chinese Medical Sciences, Beijing, China

**Keywords:** Chinese herbal formula, obesity, glucolipid metabolism disorder, adipose browning effect, thermogenesis, UCP1

## Abstract

**Background:**

Ling-gui-zhu-gan (LGZG) formula has been demonstrated to effectively ameliorate the clinical symptoms of patients with obesity or metabolic syndrome. This study aimed to explore both the effect and the underlying mechanisms of LGZG against obesity.

**Methods:**

Male C57BL/6N mice were randomized into four groups (n = 8): normal control (NC), obese (OB), metformin (Met), and LGZG. After 8 weeks of gavage administration, the pharmacological effects of LGZG on obesity and metabolism were investigated using biochemical parameters, histomorphological examination, and lipidomics techniques. Pivotal factors associated with white adipose tissue browning were evaluated using quantitative real-time polymerase chain reaction and western blotting.

**Results:**

The results revealed that LGZG reduced the levels of obesity markers, including body weights, body fat mass and food intake in obese mice. Further evaluations highlighted that LGZG restored glucose homeostasis and significantly improved insulin sensitivity in obese mice. Importantly, LGZG could adjust serum lipid profiles and regulate the lipidomic spectrum of intestinal contents, with noticeable shifts in the levels of certain lipids, particularly diacylglycerols and monoacylglycerols. Histopathological examinations of LGZG-treated mice also revealed more favorable adipose tissue structures than their obese counterparts. Furthermore, we found that LGZG upregulated the expression of several key thermogenesis-related factors, such as UCP1, PRDM16, PGC-1α, PPARα, PPARγ, CTBP1, and CTBP2 in white adipose tissues.

**Conclusion:**

Our findings position LGZG as a novel strategy for preventing obesity and improving metabolic health.

## Introduction

Obesity is a chronic metabolic disease characterized by abnormal or excessive fat accumulation due to long-term disequilibrium between calorie intake and expenditure (Fan et al., 2023). It has burgeoned into a significant global public health challenge in the 21st century, posing a striking threat to both the physical and mental health of humans. In 2016, the World Health Organization (WHO) reported that more than 1.9 billion adults were overweight, with more than 650 million among them classified as obese (WHO, 2017). Projections suggest that by 2030, approximately 2.16 billion individuals will be overweight, with about 50% progressing to obesity and its associated comorbidities (Kelly et al., 2008). Currently, obesity is the fifth principal contributor to global mortality and a prominent hazard factor in the progression of multiple chronic diseases, such as type 2 diabetes mellitus (T2DM), nonalcoholic fatty liver (NAFL), cardiovascular disease, chronic kidney disease, certain types of cancer, musculoskeletal disease, obstructive sleep apnea, and depression (Blüher, 2019; Safaei et al., 2021). Therefore, effective countermeasures and research are urgently needed to curb the development of obesity.

Adipose tissue, a pivotal regulator of whole-body energy balance, consists of white adipose tissue (WAT) and brown adipose tissue (BAT) in mammals. WAT primarily functions as an energy reservoir, storing surplus energy in the form of triglycerides (TG), whereas BAT serves as a thermogenic tissue that generates heat through non-shivering thermogenesis and promotes energy expenditure via fat oxidation (Hachemi and Mueez, 2023; Santillana et al., 2023). The mass and activity of BAT peaks during infancy and then regresses in adulthood (Wang et al., 2020). In contrast, WAT is a major type of adipose tissue in adults that is distributed in different repositories throughout the body, making it a possible target for developing new strategies for treating obesity (Wang et al., 2023).

Beige adipocytes, a new type of adipocytes detected in WAT, exhibit characteristics of both WAT and BAT, and contain a single large lipid droplet, some small lipid droplets and multiple mitochondria (Shaw et al., 2023). The emergence of beige adipocytes within WAT is defined as the “browning of WAT”, which is triggered by a sea of external stimuli, including chronic cold exposure, exercise, pharmacological interventions, and dietary factors, as well as natural compounds such as emodin, capsaicin, berberine, resveratrol, curcumin, and others (Fan et al., 2019; Wu et al., 2019; Cheng et al., 2021b; Ma et al., 2022). Although beige adipose tissue has a lower capacity for thermogenesis than the classic BAT, it contributes greatly to elevated glucose uptake, lipid decomposition, insulin sensitivity, and energy expenditure (Cheng et al., 2021a; Valdivia et al., 2023). Therefore, approaches to activate WAT browning, which may cover the inherent limitation of the scarcity of BAT mass, accounting for less than 1% of total body weight in adults, have gained significant attention as promising strategies to combat obesity and related metabolic diseases (Pilkington et al., 2021).

WAT browning involves the expression and activation of a sequence of crucial thermogenesis-related factors. Uncoupling protein 1 (UCP1), a brown fat marker protein residing within the mitochondria, could uncouple the electron transport chain from ATP synthesis to facilitate heat generation (Rahbani et al., 2024). Recent studies have confirmed that WAT browning manifested as UCP1 up-regulation can battle against obesity and hyperlipidemia, making it a promising target for obesity treatment (Chen et al., 2023; Pacifici et al., 2023). In the meantime, this process of WAT browning is associated with a set of transcriptional cascades involving PR domain containing 16 (PRDM16), peroxisome proliferator activated receptors (PPARs), and peroxisome proliferator activated receptor γ coactivator-1α (PGC-1α), and others. PRDM16 functions as a pivotal molecular switch that governs both WAT and BAT development. It not only synergizes with factors like peroxisome proliferator activated receptor α (PPARα), peroxisome proliferator activated receptor γ (PPARγ), and PGC-1α to facilitate the expression of the thermogenic factor UCP1 (Han et al., 2021; Ma et al., 2022), but also collaborates with C-terminal binding proteins 1 and 2 (CTBP1 and CTBP2), recognized as transcriptional corepressor to suppress the WAT differentiation (Lo and Sun, 2013). Thus, PRMD16 plays a central role in the WAT browning process.

Considering the global prevalence of obesity, novel interventions are required. Conventional methods, such as diet and exercise often face challenges of long-term adherence, whereas bariatric surgeries and slimming drugs have limitations owing to significant adverse effects (Aaseth et al., 2021; Chao et al., 2021). In recent years, traditional Chinese herbal medicines have been favored by the public worldwide for management of obesity and other metabolic diseases (Chen et al., 2023). Ling-gui-zhu-gan (LGZG), which consists of *Smilax glabra* Roxb. [Smilacaceae; Poria], *Neolitsea cassia* (L.) Kosterm. [Lauraceae; Cinnamomi Ramulus], *Atractylodes macrocephala* Koidz. [Asteraceae; Atractylodis macrocephalae Rhizoma], and *Glycyrrhiza glabra* L. [Fabaceae; Glycyrrhizae Radix et Rhizoma], is a classic Chinese herbal formula used to treat illnesses associated with Qi deficiency and phlegm accumulation. Research has demonstrated the reliable characteristics of LGZG in reducing body weight, decreasing blood glucose and lipids levels in individuals with obesity or metabolic syndrome in clinical trials (Luo et al., 2021; Li et al., 2023). Our preliminary research has shown that LGZG could significantly decrease body weight, body fat, and serum glucose-lipid levels, improve glucose tolerance, and relieve insulin resistance (IR) in high-fat diet (HFD)-induced diabetic mice (Wu et al., 2019), but its precise molecular mechanism remains unclear. Notably, cinnamaldehyde, a main constituent of *N. cassia* (L.) Kosterm. [Lauraceae; Cinnamomi Ramulus] in LGZG formula, can induce the browning of WAT, which motivated the current study (Neto et al., 2022). In contrast to the existing research that primarily focuses on the general effects of herbal treatments on obesity, the present study delves into the specific molecular mechanisms of adipose tissue browning. By demonstrating how LGZG stimulates factors related to adipose tissue browning and reshapes the lipid profile of the intestinal contents in obese mice, our study provides insights that could offer a novel therapeutic avenue for controlling obesity.

## Materials and methods

### Drugs

LGZG granules were purchased from Jiangyin Tianjiang Pharmaceutical Co., Ltd. (Jiangyin, China). It contained *S. glabra* Roxb. (Smilacaceae; Poria), *N. cassia* (L.) Kosterm. (Lauraceae; Cinnamomi Ramulus), *A. macrocephala* Koidz. (Asteraceae; Atractylodis macrocephalae Rhizoma), and *G. glabra* L. (Fabaceae; Glycyrrhizae Radix et Rhizoma) at a ratio of 4:3:3:2, and were prepared by a series of production processes such as extraction, separation, concentration, drying, granulation and packaging according to the “Technical Requirements for Quality Control and Standard Formulation of Chinese Medicine Formula Granules” issued by the State Food and Drug Administration (Beijing, China). The chemical constituents were characterized for quality control using high-performance liquid chromatography (HPLC), with the primary signals presented in Supplementary Figure S1. Then LGZG granules were dissolved in sterilized water for intragastric administration. Metformin hydrochloride tablets, purchased from Sino-American Shanghai Squibb Pharmaceutical Co., Ltd. (Shanghai, China), were dissolved with sterilized water before oral administration. Insulin injection was bought from Novo Nordisk (Copenhagen, Denmark).

### Reagents

Biochemistry reagent kits including TG, total cholesterol (TC), low-density lipoprotein cholesterol (LDL-C), high-density lipoprotein cholesterol (HDL-C), and non-esterified fatty acid (NEFA) were bought from Nanjing Jiancheng Bioengineering Institute (Nanjing, China). Insulin ELISA assay kit was purchased from Jiangsu Kete Biotechnology Co., Ltd. (Yancheng, China). RIPA protein lysis buffer, protease, and phosphatase inhibitors were obtained from Beijing Aoqing Biotechnology Co., Ltd. (Beijing, China). Phenylmethylsulfonyl fluoride (PMSF) was bought from Beijing Solarbio Science & Technology Co., Ltd. (Beijing, China). The BCA protein content detection kit was provided by KeyGen Biotech (Nanjing, China). The hypersensitive ECL chemiluminescence kit was bought from NCM Biotech (Suzhou, China). The polyvinylidene difluoride (PVDF) membrane was obtained from Millipore (Merck KGaA, Darmstadt, Germany). Primary antibodies to UCP1, PGC-1α, PPARα, PPARγ, CTBP1, CTBP2, and β-Tubulin (Cat no: 23673-1-AP, 66369-1-Ig, 15540-1-AP, 16643-1-AP, 10972-1-AP, 10346-1-AP, and 66240-1-lg) and secondary antibodies to rabbit and mouse (Cat no: SA00001-1 and SA00001-2) were purchased from the Proteintech Group (Chicago, IL, United States), while the primary antibody to PRDM16 (Cat no: PA5-20872) was from Invitrogen (Carlsbad, CA, United States). Trizol extraction reagent and the Revert Aid First Strand cDNA Synthesis Kit were purchased from Thermo Fisher Scientific (Waltham, MA, United States). Power SYBR Green PCR master mix was from Invitrogen (Carlsbad, CA, United States). The primer sequences for UCP1, PRDM16, PGC-1α, PPARα, PPARγ, CTBP1, CTBP2, and β-actin were synthesized by Sangon Biotech (Shanghai, China).

### Animal experimental design

Male C57BL/6N mice (8 weeks old) were purchased from Beijing Vital River Laboratory Animal Technology Co., Ltd. [Certificate No: SCXK (Beijing) 2021-0011]. The protocol was approved by the Animal Care Committee of Beijing University of Chinese Medicine (Certificate No: BUCM-1-2023012001-0025). All mice were housed in the pathogen-free barrier facility conditions: a temperature of 23°C ± 2°C, humidity maintained at 55% ± 10%, and a 12 h light/dark cycle. They were provided with free access to both diet and water. After a week of acclimation, the mice were stochastically divided into two groups, the normal control group (NC; n *=* 8) and the HFD group (HFD; n *=* 32). Mice in the NC group were fed a normal diet [20% kcal% from protein, 10% kcal% from fat, 70% kcal% from carbohydrate; SBF Biotechnology Co. Ltd., (Beijing, China)] with a caloric content of 3.1 kcal/g, while mice in the HFD group were fed a high fat diet [20% kcal% from protein, 45% kcal% from fat, 35% kcal% from carbohydrate; Medicience Biomedical Co. Ltd., (Jiangsu, China)] with a caloric content of 4.73 kcal/g for 8 consecutive weeks to induce obesity. Then the mice whose body weight were over 20% higher than that of normal mice were randomly subdivided into three groups (n *=* 8): obese group (OB), metformin group (Met, 200 mg/kg/d), and LGZG group (3.6 g/kg/d). The dosage of LGZG administered to mice was determined based on the findings from our preliminary research and by referring to the methods described in “*Pharmacological experiments methodology*” (Xu et al., 2003; Wu et al., 2019). Mice in the NC and OB groups received equivalent volume sterilized water, and all mice were administered their respective treatment via gavage for successive 8 weeks.

**FIGURE 1:**
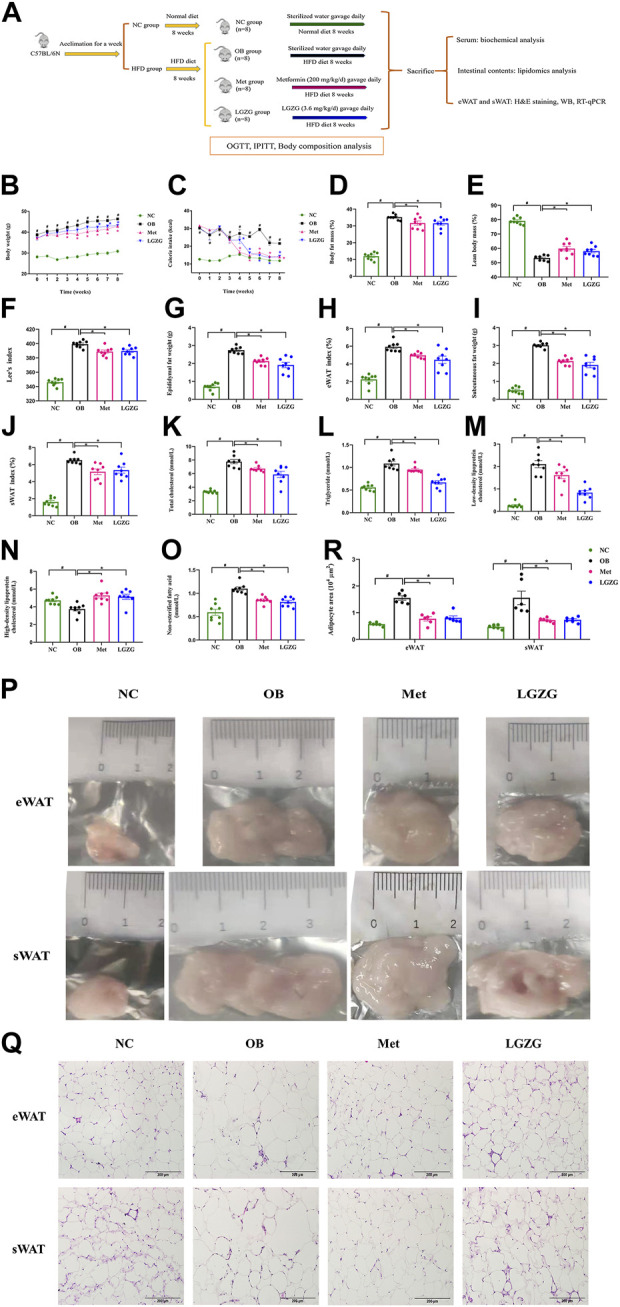
LGZG suppressed obesity and improved dyslipidemia in HFD-induced mice. **(A)** Experimental design flowchart. Comparison of **(B)** body weight and **(C)** calorie intake over administration period. **(D)** Body fat mass, **(E)** lean body mass, **(F)** Lee’s index, **(G)** epididymal fat weight, **(H)** epididymal white adipose tissue (eWAT) index, **(I)** subcutaneous fat weight, **(J)** subcutaneous white adipose tissue (sWAT) index of mice after 8 weeks of treatment. Serum **(K)** total cholesterol, **(L)** triglyceride, **(M)** low-density lipoprotein cholesterol, **(N)** high-density lipoprotein cholesterol, **(O)** non-esterified fatty acid of mice in each group. **(P)** The representative images for eWAT and sWAT of mice in each group. **(Q)** Histological sections of hematoxylin-eosin-stained-stained eWAT and sWAT at × 200 magnification. **(R)** Histograms of adipocyte area of eWAT and sWAT of mice in each group. All data were presented as mean ± SEM, with n = 8 in **(B–O)**, n = 6 in **(R)**. ^#^ *p* < 0.05 vs. the NC group, ^*^ *p* < 0.05 vs. the OB group. Time in **(B,C)** refers to weeks of administration. Calorie intake in the NC group = food intake/g × 3.1 kcal/g, calorie intake in the HFD groups = food intake/g × 4.73 kcal/g.

During the administration period, the general conditions of all mice were monitored daily, including mental state, coat luster, and activity. The body weight, fasting blood glucose (FBG), and food intake were recorded weekly. The Lee’s index was calculated using the formula: body weight 1/3 × 1,000/length (nasal-anal length in cm) (Hao et al., 2020). Body compositions were detected by the nuclear magnetic resonance body composition analyzer (Echo Medical System, Houston, TX, United States). At the end of intervention, mice were first anesthetized with 1% sodium pentobarbital to ensure they were unconscious and free from pain during the subsequent procedures. Following anesthesia, blood samples were collected from the heart for further detection of TC, TG, LDL-C, HDL-C, NEFA, and insulin levels using commercial kits according to manufacturers’ instructions. The glycosylated hemoglobin (HbA1c) was quantified using an automatic HbA1c analyzer (Yangzhou Medik Medical Technology Co., Ltd., Yangzhou, China). After the completion of blood sample collections, all mice were euthanized by cervical dislocation. This method was selected for its ability to quickly and humanely terminate the mice, following the ethical guidelines for animal research. Epididymal WAT (eWAT) and subcutaneous WAT (sWAT) were promptly harvested and weighed. Then portions of eWAT and sWAT were fixed in 4% paraformaldehyde, while the remainder was stored at −80°C refrigerator for subsequent experiments. The intestinal contents from five randomly selected mice in each group were collected for lipidomics analysis. A schematic representation of the experimental design is depicted in Figure 1A below.

### Oral glucose tolerance test (OGTT) and intraperitoneal insulin tolerance test (IPITT)

In OGTT and IPITT, mice were administered glucose (2 g/kg body weight, P.O.) and insulin (0.5 U/kg body weight, I.P.), respectively. Blood glucose levels from the tail vein were measured before and at 30, 60, 90, and 120 min after administration of glucose or insulin using a blood glucose meter (Johnson & Johnson, New Brunswick, NJ, United States). The area under the curve (AUC) of the glucose levels was calculated.

### Histomorphology analysis

The fixed eWAT and sWAT samples were processed following the routine steps of rinsing in water overnight, dehydrating in a gradient of alcohol concentration from 75% to 100%, and paraffin embedding. The paraffin sections, sliced to 5 μm thickness, were subjected to hematoxylin-eosin (H&E) staining and examined under an inverted microscope (Olympus Corporation, Tokyo, Japan) for estimating the histopathological changes in eWAT and sWAT.

### Absolute quantitative lipidomics

Lipidomics analysis was conducted in collaboration with Shanghai Applied Protein Technology Co., Ltd. Lipids from intestinal contents were analyzed based on methyl tert-butyl ether (MTBE) method, as previously described according to the reference (Yao et al., 2021). The lipids were extracted from the samples and reconstituted with 200 μL of 90% isopropanol/acetonitrile and then centrifuged. Subsequently, 90 μL of supernatant was injected into a CSH C18 column (1.7 μm, 2.1 mm × 100 mm) at a flow rate of 500 μL/min using an UHPLC Nexera LC-30A ultra performance liquid chromatography system (SHIMADZU, Kyoto, Japan). The analytes were eluted by mobile phases A (acetonitrile:water = 6:4) and mobile phases B (acetonitrile: isopropanol = 9:1). The mass spectrographic analysis was performed in positive and negative heated electrospray ionization (ESI) using a Q exactive mass spectrometer (Thermo Fisher Scientific, Waltham, MA, United States). The Lipidsearch software (Thermo Fisher Scientific, Waltham, MA, United States) was used to analyze the data in positive and negative ion modes. Quality control (QC) samples were set to evaluate and monitor instrument stability and experiment repeatability throughout the analysis. For lipidomics analysis, multivariate unsupervised principal component analysis (PCA) was performed using the scatterplot3d package. This involved preprocessing the data, computing the covariance matrix, calculating eigenvalues and eigenvectors, selecting principal components, and projecting the data onto these components. The results were visualized using a scatter plot to identify sample clusters and interpret the similarities and differences in lipid profiles.

### Reverse transcription quantitative real-time polymerase chain reaction (RT-qPCR) analysis

Total RNA was extracted from eWAT and sWAT with Trizol reagent, then it was quantified by a nanophotometer^®^ all-in-one spectrophotometer (IMPLEN, Munich, Germany) and reverse transcripted into cDNA by the Reverse Transcription System (Bio-Rad, Hercules, CA, United States). qPCR analysis was carried out to determine the expression levels of the target genes including UCP1, PRDM16, PGC-1α, PPARα, PPARγ, CTBP1, and CTBP2 using CFX qPCR Detection System (Bio-Rad, Hercules, CA, United States) with the following procedures: all samples were pre-denatured at 95°C for 10 min, then followed by 40 cycles of amplification at 95°C for 15 s and 60°C for 45 s, and finally the melting curve was generated. The relative gene expression values were normalized to the β-actin according to the 2^-△△Ct^ method. The sequences of primers in this study are available in Table 1.

**TABLE 1:** Primer sequences used for RT-qPCR experiments.

Gene name	Forward (5′-3′)	Reverse (5′-3′)
UCP1	GAA​ACA​CCT​GCC​TCT​CTC​GGA​AAC	GCA​TTC​TGA​CCT​TCA​CGA​CCT​CTG
PRDM16	CAG​CAA​CCT​CCA​GCG​TCA​CAT​C	GCG​AAG​GTC​TTG​CCA​CAG​TCA​G
PGC-1α	CGA​TGA​CCC​TCC​TCA​CAC​CAA​AC	TTG​CGA​CTG​CGG​TTG​TGT​ATG​G
PPARα	CTT​CAC​GAT​GCT​GTC​CTC​CTT​GAT​G	GAT​GTC​ACA​GAA​CGG​CTT​CCT​CAG
PPARγ	GCC​AAG​GTG​CTC​CAG​AAG​ATG​AC	GTG​AAG​GCT​CAT​GTC​TGT​CTC​TGT​C
CTBP1	CAG​TGA​GCA​GGC​GTC​CAT​TGA​G	GGC​TGT​CAG​GTG​GTC​CTT​GTT​G
CTBP2	TGA​GAA​GGT​GTT​GAA​TGA​GGC​TGT​G	CAC​TAC​CGA​TTC​GCA​CGA​TCA​CTC

### Western blot (WB) analysis

eWAT and sWAT were lysed with RIPA lysate buffer and relevant enzyme inhibitors for protein extracting, and then the protein concentrations were determined using a BCA protein assay kit. A WB electrophoresis apparatus (Bio-Rad, Hercules, CA, United States) was applied for protein analysis. The complete experimental procedures included electrophoresis, membrane transfer, blocking, and incubation with primary and secondary antibodies. The antibodies used in this study were diluted at the following dilutions: anti-UCP1, anti-PRDM16, anti-CTBP1, and anti-CTBP2 at 1:1,000; anti-PGC-1α and anti-PPARγ at 1:2,000; anti-PPARα at 1:500; anti-β-tubulin at 1:20,000; and secondary antibodies to rabbit and mouse at 1:5,000. Finally, the objective protein bands were photographed using a gel imager (Clinx, Shanghai, China), and the relative expressions of the target proteins were analyzed with the Image J software package.

### Statistical analysis

According to the Shapiro-Wilk test, the normality of data distribution was assessed. For normally distributed data, the comparison among groups was performed adopting one-way or two-way analysis of variance (ANOVA), followed by Dunnett’s multiple comparison test with GraphPad prism 7 software. All data were expressed as mean ± standard error of the mean (SEM). Differences were considered statistically significant at *p* < 0.05.

## Results

### LGZG ameliorated HFD-induced obesity and dyslipidemia

A HFD-induced obesity model was established to investigate the effects of LGZG on obesity. Compared with mice fed a normal diet, obese mice displayed relatively dull fur luster, diminished energy, and reduced activity levels. These conditions were improved notably after 8 weeks of LGZG administration. As depicted in Figures 1B–J, the HFD-fed mice exhibited a remarkable increase in body weight, calorie intake, body fat mass, Lee’s index, as well as the weight and index of eWAT and sWAT compared to the normal mice (*p* < 0.05). After an 8-week treatment period, LGZG, along with metformin, significantly decreased the body weight of obese mice (Figure 1B, *p* < 0.05) and effectively lowered the body fat mass, Lee’s index, and the weight and index of eWAT and sWAT, while significantly increasing lean body mass (Figures 1D–J, *p* < 0.05). The calorie intake results shown in Figure 1C indicated that both metformin and LGZG had a significant effect on restricting the calorie intake of obese mice from week 4 onwards (*p* < 0.05).

To investigate the therapeutic potential of LGZG in lipid metabolic disorders in obese mice, the serum levels of TC, TG, LDL-C, HDL-C, and NEFA were measured. As shown in Figures 1K–O, the OB group displayed a significant increase in the levels of TC, TG, LDL-C, and NEFA, along with a remarkable decrease in the level of HDL-C, compared with the NC group (*p* < 0.05). This suggests that HFD-induced dyslipidemia occurred in obese mice, whereas metformin and LGZG ameliorated these lipid disturbances to different degrees (*p* < 0.05). The correlation between visceral and subcutaneous fat and metabolic diseases is well-documented (Rosen and Spiegelman, 2014; Chen et al., 2021). Therefore, the histopathological examination of eWAT and sWAT in each group was performed using H&E staining. We observed that eWAT and sWAT exposed to LGZG were smaller in size (Figure 1P). Furthermore, adipocytes within the eWAT and sWAT of mice receiving LGZG treatment appeared smaller, more densely arranged, and displayed distinct nuclei compared to those in the OB group (Figures 1Q, R). Collectively, these results indicated that LGZG effectively relieved HFD-induced obesity and dyslipidemia in obese mice.

### LGZG maintained glucose homeostasis and ameliorated IR in obese mice

**FIGURE 2:**
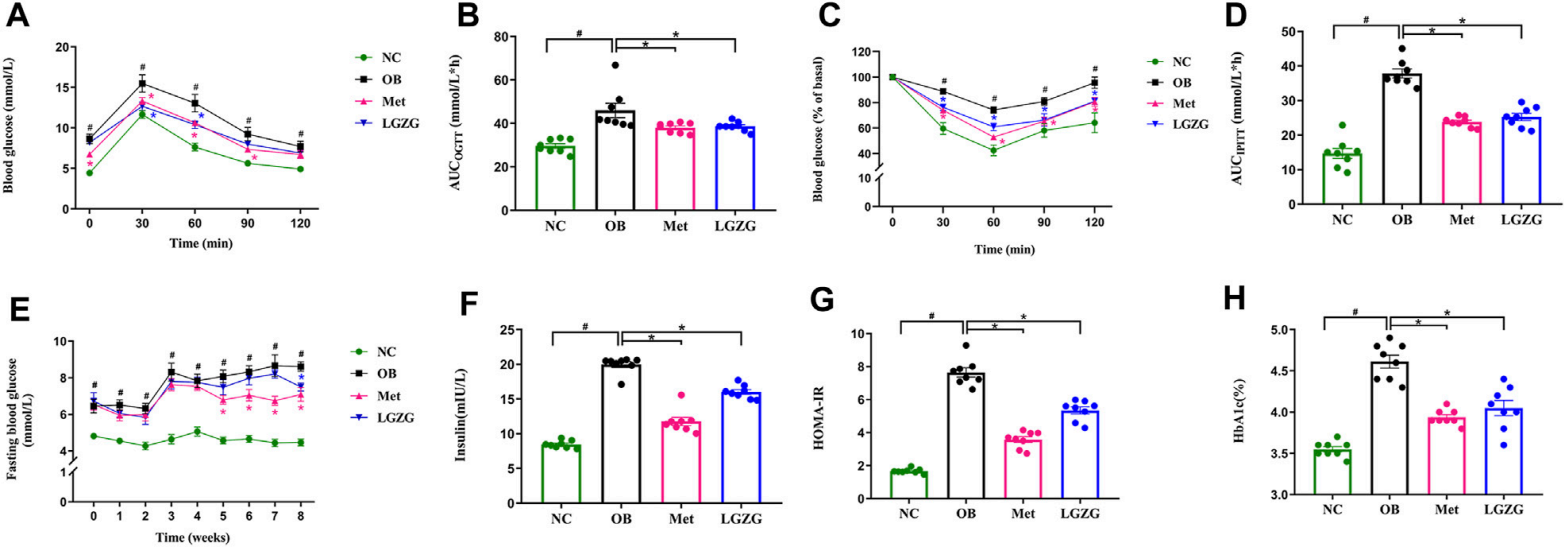
LGZG ameliorated the disordered glucose metabolism and IR in obese mice. **(A)** Blood glucose level over time in OGTT. **(B)** Area under the curve (AUC_OGTT_). **(C)** Blood glucose level over time in IPITT. **(D)** Area under the curve (AUC_IPITT_). **(E)** Fasting blood glucose levels and **(F)** fasting serum insulin levels. **(G)** Homeostasis model assessment of insulin resistance (HOMA-IR). **(H)** Glycosylated hemoglobin. All data were presented as mean ± SEM, with n = 8 in **(A–H)**. ^#^ *p* < 0.05 vs. the NC group, ^*^ *p* < 0.05 vs. the OB group. AUC was calculated as follows (Leng et al., 2014): AUC (mmol/L × h) = 0.5 h × (BG0 + BG30 min)/2 + 0.5 h × (BG30 + BG60 min)/2 + 1 h × (BG60 + BG120 min)/2, where BG refers to blood glucose. Time in **(E)** refers to weeks of administration. HOMA-IR was calculated by multiplying FBG (mmol/L) and fasting insulin (mU/L) divided by 22.5 (Wu et al., 2022).

In a previous study, we reported that LGZG improved disordered glucose metabolism in diabetic mice (Wu et al., 2019). In this study, we validated the effect of LGZG on glucose homeostasis and insulin sensitivity in obese mice using OGTT and IPITT. The OGTT results (Figures 2A, B) revealed impaired glucose tolerance in the OB group, as evidenced by a notably increased AUC compared to the normal mice (AUC of NC and OB groups: 29.61 ± 1.10 and 45.97 ± 3.32, respectively, *p* < 0.05). The glucose levels of the Met and LGZG groups were significantly lower than that of the OB group at 30 and 60 min during the OGTT (*p* < 0.05), along with obviously lower AUC values (AUC of Met and LGZG groups: 38.06 ± 0.83 and 38.65 ± 0.78, respectively).

IR is intricately linked to obesity progression, and is an essential pathological change in the development of T2DM among individuals with obesity (Tong et al., 2022). Therefore, we conducted an IPITT, assessed fasting insulin levels, and calculated the homeostasis model assessment of insulin resistance (HOMA-IR, Figures 2C, D, F, G). Metformin and LGZG exhibited a remarkable increase in insulin-induced glucose utilization compared to the OB group (*p* < 0.05), which revealed that both metformin and LGZG could effectively enhance insulin sensitivity in obese mice. Accordingly, LGZG treatment reduced serum insulin levels, which tended to increase in obesity (*p* < 0.05), and significantly reduced HOMA-IR in comparison with the OB group (*p* < 0.05). Notably, LGZG could also significantly reduce FBG, which was consistent with our preliminary study (Figure 2E, *p* < 0.05), although this hypoglycemic effect may take several weeks to fully manifest. Additionally, a lower value of HbA1c, the key index for monitoring long-term blood glucose levels, was observed in the LGZG treated mice than that in their untreated counterparts (Figure 2H, *p* < 0.05). In general, these findings demonstrate the capacity of LGZG to counteract disrupted glucose metabolism and improve IR, thus holding promise as a potential therapeutic intervention for obesity.

### LGZG altered the lipid metabolic profiles of intestinal contents in HFD-induced obese mice

**FIGURE 3:**
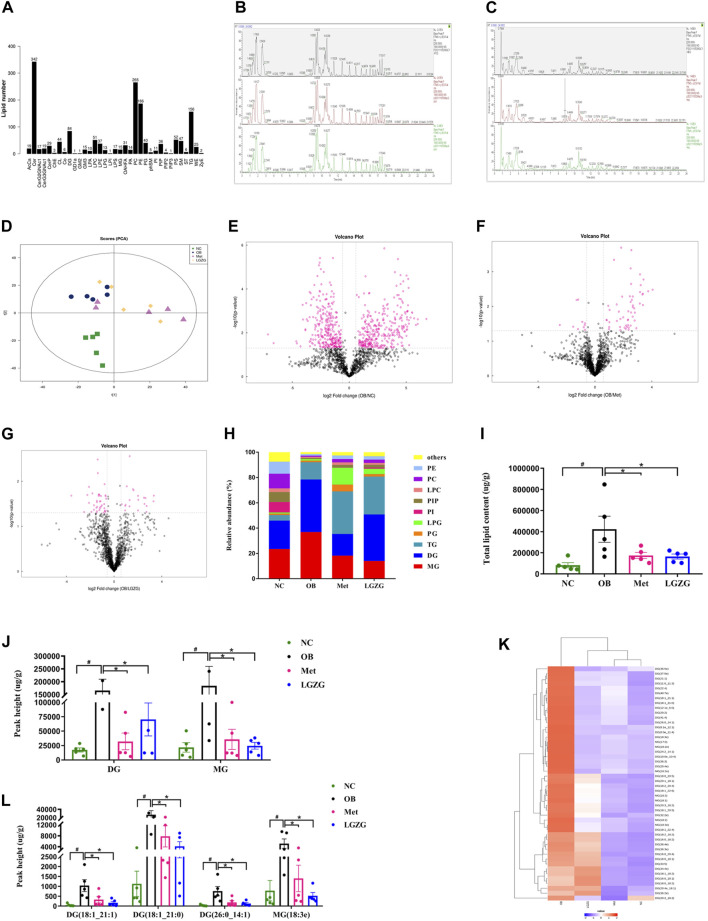
Overview of the LGZG’s impact on lipid profiles in the intestinal contents of obese mice. **(A)** Lipid classes and the number of lipid species contained in each class. The base peak spectrogram overlaps spectra of quality control (QC) samples in **(B)** positive and **(C)** negative ion mode. **(D)** Principal component analysis (PCA) score plot (*R* ^2^ *X* = 0.516). The volcano plot highlighted the trend of lipid species between **(E)** OB and NC groups; **(F)** OB and Met groups; **(G)** OB and LGZG groups. Rose-red dots represent lipids with significant differences between groups (FC > 1.5 or <0.67, *p* < 0.05). **(H)** Relative abundance of the lipid composition at the class level. **(I)** Sum of all identified lipid species in mice intestinal contents. **(J)** Amount of DG and MG classes in the intestinal contents. **(K)** Heat map of lipid species in the DG and MG categories with FC > 1.5 or < 0.67 and *p* < 0.05. **(L)** Contents of lipid species including DG (18:1_21:1), DG (18:1_21:0), DG (26:0_14:1), and MG (18:3e) in the intestinal contents. All data were presented as mean ± SEM, with *n* = 5 in **(A–L)**. ^#^ *p* < 0.05 vs. the NC group, ^*^ *p* < 0.05 vs. the OB group. DG, diglyceride; MG, monoglyceride.

Based on the experimental results, we analyzed the lipid profiles of the intestinal contents using non-target metabolomics. A total of 1,605 lipid molecular species were identified from the intestinal contents of mice using UPLC-MS/MS, covering 35 lipid classes including acyl carnitine (AcCa), (O-acyl)-1-hydroxy fatty acid (OAHFA), wax esters (WE), cardiolipin (CL), lysophosphatidic acid (LPA), lysophosphatidylcholine (LPC), lysophosphatidylethanolamine (LPE), lysophosphatidylglycerol (LPG), lysophosphatidylinositol (LPI), lysophosphatidylserine (LPS), phosphatidic acid (PA), phosphatidylcholine (PC), phosphatidylethanolamine (PE), phosphatidylglycerol (PG), phosphatidylinositol (PI), phosphatidylinositol (4) phosphate (PIP), phosphatidylinositol (4,5) bisphosphate (PIP2), phosphatidylinositol (3,4,5) triphosphate (PIP3), phosphatidylserine (PS), coenzyme Q (Co), monoglyceride (MG), diglyceride (DG), triglyceride (TG), zymosterol (ZyE), cholesterol ester (ChE), ceramide (Cer), N-acetylhexosyl ceramide (CerG2GNAc1), dihexosyl N-acetylhexosyl ceramide (CerG3GNAc1), ceramides phosphate (CerP), phytosphingosine (phSM), sphingomyelin (SM), sulfatide (ST), as well as ganglioside GD1a, GM2, and GM3 (Figure 3A). The stability and repeatability of the experiment were assessed by comparing the base peak spectrograms under positive and negative ion modes obtained from the mass spectrometry detection and QC samples (Figures 3B, C). The results confirmed that the chromatographic peak response intensity and retention time of each QC sample were highly consistent, indicating excellent stability and reproducibility of the experimental data, which could be used for subsequent analysis. Multivariate statistical analysis was conducted to compare the lipid profiles of the intestinal contents among groups. As shown in Figure 3D, the PCA illustrated an obvious separation between the NC and OB groups, suggesting that HFD could cause significant abnormal lipid metabolism. Interestingly, the treatment groups (Met and LGZG) showed some overlap with the OB group, indicating that LGZG intervention may partially mitigate dyslipidemia associated with obesity. The trends in lipid species between the OB group and the other three groups were clearly depicted in the volcano plots (Figures 3E–G) for the screening and analysis of differential lipids among the groups.

The lipid composition at the class level was represented by the cumulative column chart in Figure 3H. The data showed a noticeable increase in the total levels of the lipid species identified in the intestinal contents of the model mice compared to the other three groups (Figure 3I, *p* < 0.05). Among these changes, DG and MG contributed the most, and their levels were significantly lower in the NC, Met, and LGZG groups than in the OB group (Figure 3J, *p* < 0.05). Next, to identify the specific lipid species responsible for the variation within the DG and MG classes among the four groups, a criterion of fold change (FC) > 1.5 or < 0.67, coupled with *p* < 0.05 was used. Specifically, we found that 22 of DG and 6 of MG lipid species exhibited significant differences in the NC group compared to the OB group. In the Met group, 41 DG and 9 MG lipid species showed significant differences, while in the LGZG group, 7 DG and 1 MG lipid species exhibited significant differences compared to the OB group. Notably, the levels of these distinct lipid molecules were markedly increased in the HFD-induced obese mice. Hierarchical clustering and a heat map were generated to visualize the abundance of these selected different metabolites (Figure 3K). Among them, DG (18:1_21:1), DG (18:1_21:0), DG (26:0_14:1) and MG (18:3e) were perceived as commonly distinct metabolites in the three comparisons (OB vs. NC, OB vs. Met, and OB vs. LGZG), and the peak values of these lipid species in the intestinal contents of the mice in the OB group were significantly higher than those observed in the other three groups (Figure 3L, *p* < 0.05).

### LGZG facilitated browning of WAT in HFD-induced obese mice

**FIGURE 4:**
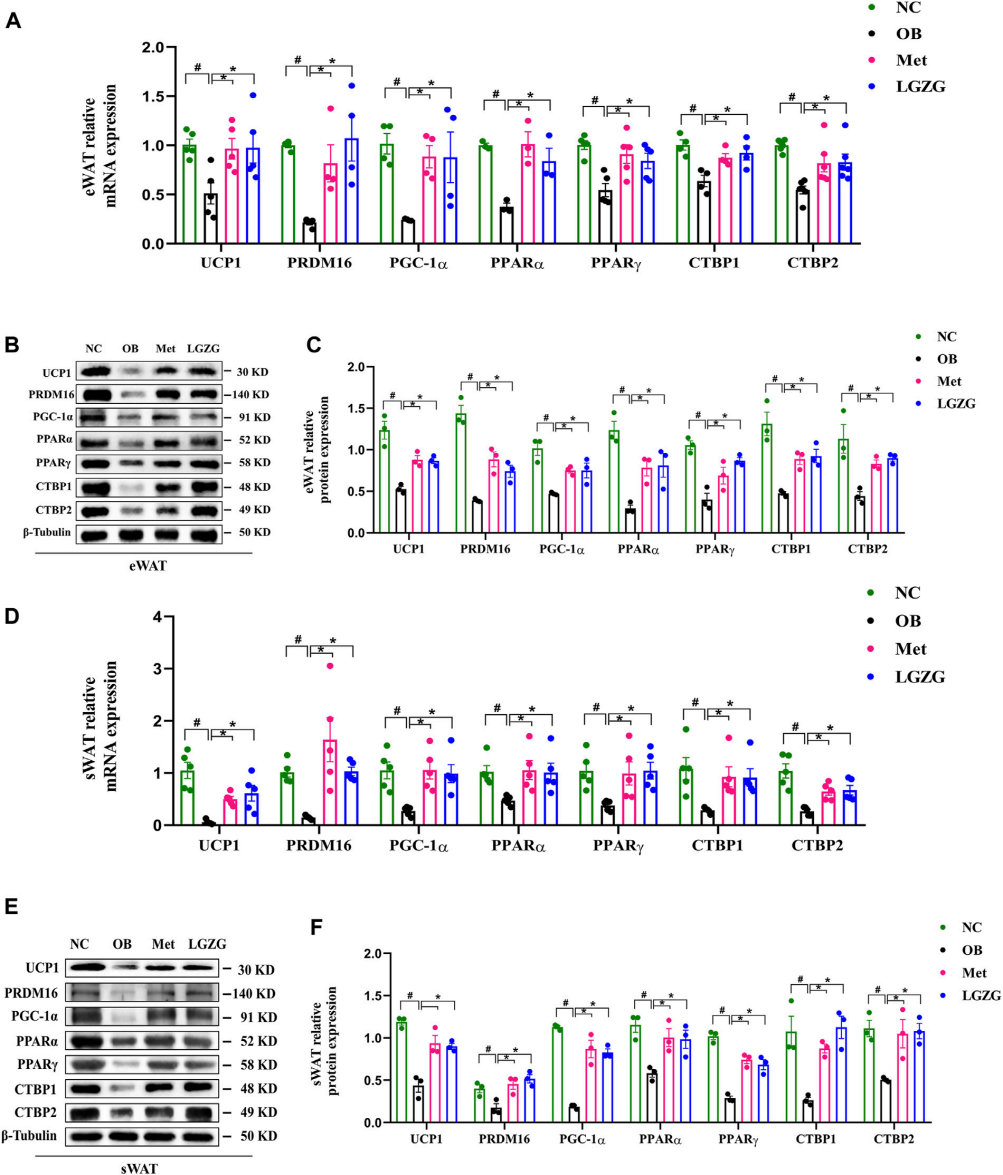
Effect of LGZG on the expression of thermogenesis-related genes and proteins in HFD-induced obese mice. **(A)** Relative genes expressions of UCP1, PRDM16, PGC-1α, PPARα, PPARγ, CTBP1, and CTBP2 in eWAT. **(B,C)** The image analysis and quantitative analysis results of proteins to UCP1, PRDM16, PGC-1α, PPARα, PPARγ, CTBP1, and CTBP2 in eWAT. **(D)** Relative genes expressions of UCP1, PRDM16, PGC-1α, PPARα, PPARγ, CTBP1, and CTBP2 in sWAT. **(E,F)** The image analysis and quantitative analysis results of proteins to UCP1, PRDM16, PGC-1α, PPARα, PPARγ, CTBP1, and CTBP2 in sWAT. All data were presented as mean ± SEM, with *n* = 3∼6 in **(A)**, *n* = 5 in **(D)**, n = 3 in **(B,C,E,F)**. ^#^ *p* < 0.05 vs. the NC group, *p < 0.05 vs. the OB group. UCP1, uncoupling protein 1; PRDM16, PR domain containing 16; PGC-1α, peroxisome proliferator-activated receptor γ coactivator-1α; PPARα, peroxisome proliferator activated receptor α; PPARγ, peroxisome proliferator activated receptor γ; CTBP1, C-terminal binding proteins 1; CTBP2, C-terminal binding proteins 2; eWAT, epididymal white adipose tissue; sWAT, subcutaneous white adipose tissue.

It has been previously demonstrated that inducing WAT browning could promote energy expenditure by increasing heat production, reducing adiposity, and shielding mice from HFD-induced obesity and its associated metabolic disorders. This motivated us to investigate the influence of LGZG on WAT browning. To this end, the gene and protein expressions of thermogenic factors, as well as other representative transcriptional regulators and coregulators in eWAT and sWAT, were detected using RT-qPCR and WB. As depicted in Figures 4A–F, as compared with the normal group, the expression of UCP1, a hallmark factor for WAT browning, was far less in the eWAT and sWAT of OB group, along with the decreased expression of a series of major modulators involved in WAT browning process such as PRDM16, PGC-1α, PPARα, PPARγ, CTBP1, and CTBP2 (*p* < 0.05). As expected, treatment with metformin or LGZG enhanced the proteins and genes expression of these thermogenesis-related factors (*p* < 0.05). In conclusion, LGZG exhibited considerable capacity to ameliorate metabolic disorders associated with HFD-induced obesity by inducing WAT browning.

## Discussion

Obesity arises from the interplay between genetic and environmental factors and has become a global concern. Traditional Chinese herbal medicines, known for their multi-active constituents and multi-target effects, have been widely used clinically to combat obesity because of their efficacy in complex diseases with minimal side effects (Lyu et al., 2022). LGZG, a classic prescription for invigorating the spleen function and dispelling dampness, has shown promise in the prevention and treatment of obesity in clinical practice (Luo et al., 2021; Li et al., 2023). We previously found that LGZG exerted positive effects on obesity control and the improvement of glycolipid metabolism disorders. Here, we elucidate the impact of LGZG on obesity and its related metabolic disorders, as well as uncover the underlying mechanism behind these effects.

The findings of our study demonstrate the significant hypoglycemic and weight-lowering effects of LGZG in obese mice. IR is a common metabolic disorder in obesity that leads to hyperinsulinemia and increased FBG levels and can be evaluated using HOMA-IR and IPITT (Stefan, 2020; Mocciaro and Gastaldelli, 2022). We found that mice in the OB group had higher levels of FBG and HbA1c, accompanied by impaired glucose tolerance and insulin sensitivity as well. Nevertheless, LGZG treatment effectively reversed these abnormalities, indicating that the possible mechanisms of LGZG for glycemic control may be related to its capacity to enhance insulin sensitivity. As we all know, prolonged consumption of HFD typically leads to weight gain and subsequent obesity. In the present study, LGZG reduced the body weight, body fat mass, epididymal and subcutaneous fat weight, and adipocyte size of WAT in HFD-induced obese mice. The weight-lowering effect of LGZG may be associated with the decreased calorie intake, as well as epididymal and subcutaneous fat weight. Obesity is characterized by excessive lipid accumulation in WAT arising from both adipocyte hypertrophy and proliferation, which is in line with the findings of our study to some degree (Jin et al., 2022).

Obesity is a risk factor for the development of diabetes, cardiovascular disease, and some types of cancer, and is attributed to its accompanying dyslipidemia (Sulumer et al., 2024). The typical dyslipidemia seen in obesity is characterized by elevated TC, TG, and NEFA levels, as well as high levels of LDL-C and low levels of HDL-C (Klop et al., 2013). Under normal circumstances, adipocytes regulate energy metabolism by storing energy in the form of TG and releasing NEFA, while in the obese state, adipose tissues dysfunction may arise when excessive circulating NEFA stemming from the increased basal lipolysis fluxes into adipocytes that far exceeds their storage capacity. This overflow of NEFA may ultimately lead to the development of hypertriglyceridemia and IR in peripheral organs (Park et al., 2021). Additionally, elevated TG levels are generally accompanied by decreased HDL-C levels, with the latter having a strong negative correlation with obesity (Stadler and Marsche, 2020; Nussbaumerova and Rosolova, 2023). In accordance with these findings, we analyzed the serum lipid profiles and found that treatment with metformin or LGZG in obese mice alleviated HFD-induced dyslipidemia, as evidenced by lowered concentrations of circulating NEFA, TC, TG, and LDL-C, as well as elevated levels of HDL-C.

Subsequently, our analysis extended to the lipid profiles of the intestinal contents of the mice. The lipidomics spectrum of mice was previously reported to be reshaped by HFD (Shon et al., 2020; Cheng et al., 2021b), which was reconfirmed in our study that HFD significantly increased the total lipid content in the intestinal contents of mice, particularly in terms of DG and MG. Energy excess, as the chief cause for obesity, is associated with mitochondrial dysfunction, which may lead to the accumulation of metabolic intermediates such as DG in tissues (Gjermeni et al., 2021). Abnormal accumulation of DG, a direct precursor of TAG, may boost the development of IR by disrupting insulin signaling transduction, thereby impairing insulin-stimulated glucose metabolism (Jani et al., 2021; Getiye et al., 2022). MG appears to function as an intracellular signaling mediator in TG synthesis (Mansbach and Siddiqi, 2010). Our findings indicated that LGZG treatment reversed the high levels of DG and MG classes observed in obese mice. The reshaping of the lipid profile of the intestinal contents in obese mice treated with LGZG suggests that LGZG not only improves lipid metabolism but also reduces the accumulation of harmful metabolic intermediates, thereby potentially alleviating IR and improving overall metabolic health. Special attention should be paid to the molecular pathways involved in LGZG-induced lipid metabolism. Exploring the influence of LGZG on these pathways may reveal its therapeutic mechanisms. For instance, the upregulation of UCP1, PRDM16, and PGC-1α in WAT indicates a shift towards increased thermogenesis and energy expenditure.

As we know, obesity stems from an imbalance between energy intake and expenditure. Consequently, increasing energy expenditure is perceived as a potential strategy to combat obesity (Jung et al., 2022). Induction of WAT browning offers a novel avenue for increasing energy expenditure by increasing heat generation. As expected, our further study revealed that LGZG treatment resulted in a significant up-regulation of UCP1, which is a dominant regulator of thermogenesis. The upregulation of UCP1 is considered the sign of the conversion of WAT into BAT (Li and Tang, 2021). This conversion augments energy expenditure, thereby preventing HFD-induced obesity (Liu et al., 2021). Typically, an increase in UCP1 protein levels corresponds to an increase in PRDM16 expression, which is a key determinant of BAT and beige adipocytes development (Jiang et al., 2022). In transgenic mice with PRDM16, there was an increased generation of brown-like adipocytes within WAT, leading to an improvement in obesity and abnormal glucose tolerance induced by HFD (Seale et al., 2011). Conversely, adipose PRDM16 knockdown in rodents resulted in the decreased expression of thermogenesis-related genes (Cohen et al., 2014). In addition, UCP1 activation involves the expression of PGC-1α, which plays a central role in mediating mitochondrial biogenesis, thermogenesis, and fatty-acid oxidation. PGC-1α is also acknowledged as a marker of beige adipocytes appearing in WAT, and collaborates with PRDM16 to activating thermogenic genes (He et al., 2018). Here, we proved that LGZG reduced body weight, fat mass, and Lee’s index of HFD-induced obese mice, which may benefit from increased expression of UCP1, PRDM16, and PGC-1α.

Furthermore, the transcription factors PPARγ and PPARα are pivotal components in the WAT browning cascade. PPARγ is indispensable for WAT browning, and treatment with its agonist rosiglitazone has been shown to significantly increase UCP1 expression in the WAT of both human and mice (Zhu et al., 2020; Zhang et al., 2023). Deacetylation of PPARγ facilitates synergy between PRDM16 and PPARγ, subsequently increasing the expression of browning genes like UCP1 and PGC-1α (Zhang et al., 2018). PPARα, a master regulator of lipid metabolism, also contributes to adipocyte browning by elevating UCP1 and PGC-1α levels, thus reducing body weight (Rachid et al., 2018). The PPARα-knockout obese mice fed on a HFD showed a significant elevation in visceral fat and a decrease in BAT (Haluzik et al., 2004). Additionally, PPARα potentially activates PRDM16 and collaborates with it to enhance PGC-1α, thereby promoting a BAT-like phenotype in WAT (Hondares et al., 2011).

**FIGURE 5:**
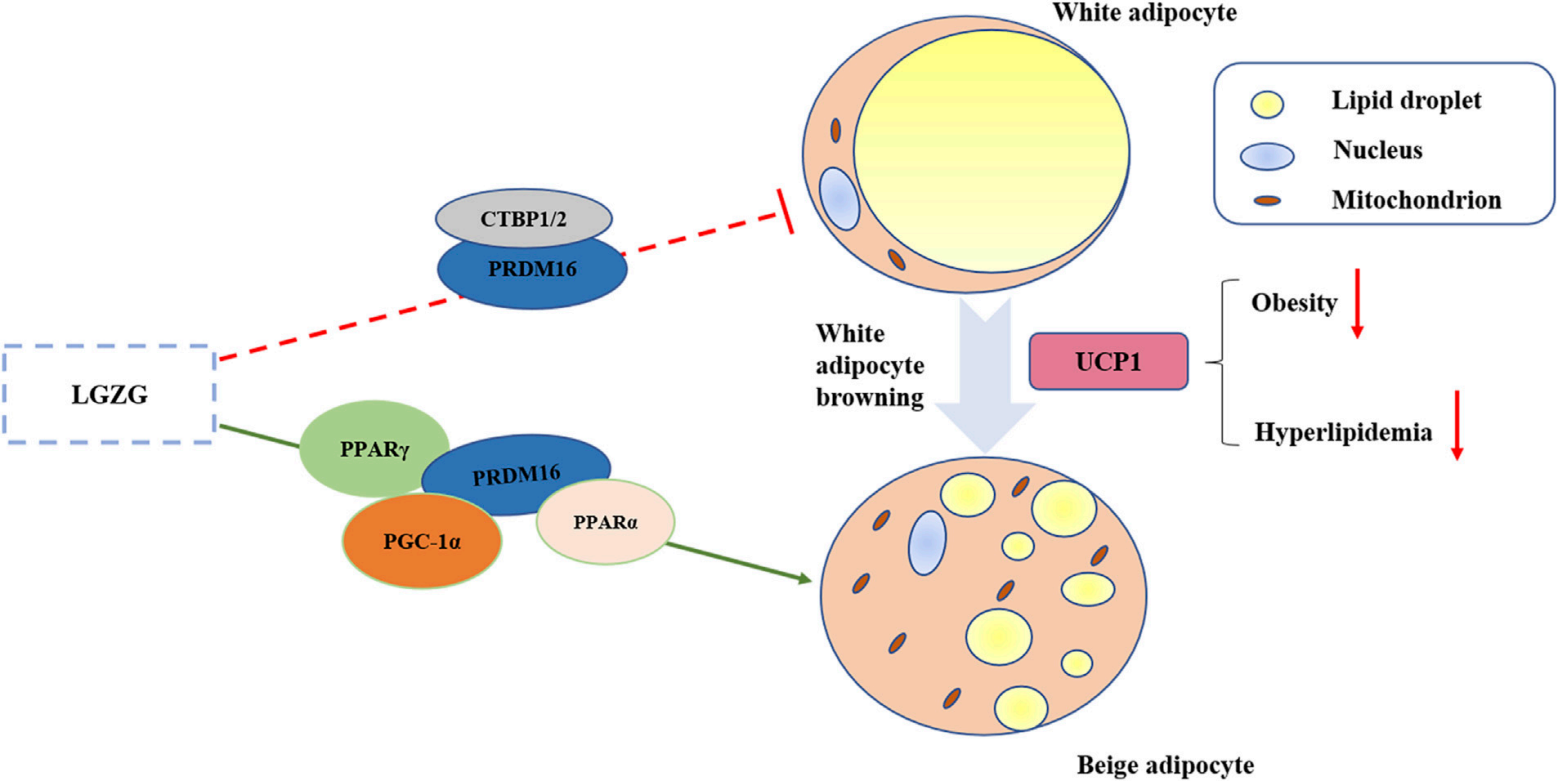
Graphical abstract of LGZG granules ameliorated metabolic disorder via stimulating white adipocytes browning.

Notably, our results showed that LGZG increased the expression of CTBP1 and CTBP2, crucial transcriptional coregulators in adipose tissue, which may regulate the conversion of WAT to BAT in collaboration with PRDM16 (Jack et al., 2011). The browning process not only involves the activation of brown adipocyte genes, but also the inhibition of white adipocyte genes. In addition to stimulating the brown adipocyte-specific genes, PRDM16 represses white adipocyte genes, which require CTBP1 and CTBP2 (Lo and Sun, 2013). To sum up, our study indicated that LGZG could activate the browning of WAT via up-regulating the expressions of UCP1, PRDM16, PGC-1α, PPARα, PPARγ, CTBP1, and CTBP2 (Figure 5). Moreover, validating these results in PRDM16-knockdown mice would provide more convincing evidence of LGZG’s efficacy and could pave the way for future clinical applications.

## Conclusion

We provide evidence that LGZG treatment has the potential to ameliorate HFD-induced obesity and metabolic disorders. This is evident from the decreased body weight and fat accumulation, improved glucose tolerance and insulin sensitivity, and correction of dyslipidemia. Notably, our investigation reveals a novel role for LGZG in stimulating WAT browning, which significantly contributes to its weight-reducing and metabolism-regulating effects. These findings highlight the multifaceted benefits of LGZG and its potential as a remedy for obesity and associated metabolic complications.

## Figures and Tables

**Table:** 

**AcCa**	Acyl carnitine
**ANOVA**	Analysis of variance
**AUC**	Area under the curve
**BAT**	Brown adipose tissue
**Cer**	Ceramide
**CerG2GNAc1**	N-acetylhexosyl ceramide
**CerG3GNAc1**	Dihexosyl N-acetylhexosyl ceramide
**CerP**	Ceramides phosphate
**ChE**	Cholesterol este
**CL**	Cardiolipin
**Co**	Coenzyme Q
**CTBP1**	C-terminal binding protein 1
**CTBP2**	C-terminal binding protein 2
**DG**	Diglyceride
**eWAT**	Epididymal white adipose tissue
**ESI**	Electrospray ionization
**FBG**	Fasting blood glucose
**HbA1c**	Glycosylated hemoglobin
**HDL-C**	High-density lipoprotein cholesterol
**H&E**	Hematoxylin-eosin
**HFD**	High-fat diet
**HPLC**	High-performance liquid chromatography
**IPITT**	Intraperitoneal insulin tolerance test
**IR**	Insulin resistance
**LDL-C**	Low-density lipoprotein cholesterol
**LGZG**	Ling-gui-zhu-gan
**LPA**	Lysophosphatidic acid
**LPC**	Lysophosphatidylcholine
**LPE**	Lysophosphatidylethanolamine
**LPG**	Lysophosphatidylglycerol
**LPI**	Lysophosphatidylinositol
**LPS**	Lysophosphatidylserine
**MG**	Monoglyceride
**MTBE**	Methyl tert-butyl ether
**NAFL**	Nonalcoholic fatty liver
**NC**	Normal control
**NEFA**	Non-esterified fatty acid
**OAHFA**	(O-acyl)-1-hydroxy fatty acid
**OB**	Obese
**Met**	Metformin
**OGTT**	Oral glucose tolerance test
**PA**	Phosphatidic acid
**PC**	Phosphatidylcholine
**PCA**	Principal component analysis
**PE**	Phosphatidylethanolamine
**PG**	Phosphatidylglycerol
**PGC-1α**	Peroxisome proliferator activated receptor γ coactivator-1α
**phSM**	Phytosphingosine
**PI**	Phosphatidylinositol
**PIP**	Phosphatidylinositol (4) phosphate
**PIP2**	Phosphatidylinositol (4,5) bisphosphate
**PIP3**	Phosphatidylinositol (3,4,5) triphosphate
**PPARα**	Peroxisome proliferator activated receptor α
**PPARs**	Peroxisome proliferator activated receptors
**PPARγ**	Peroxisome proliferator activated receptor γ
**PRDM16**	PR domain containing 16
**PS**	Phosphatidylserine
**QC**	Quality control
**RT-qPCR**	Reverse transcription quantitative real-time polymerase chain reaction
**SEM**	Standard error of mean
**SM**	Sphingomyelin
**ST**	Sulfatide
**sWAT**	subcutaneous white adipose tissue
**T2DM**	Type 2 diabetes mellitus
**TC**	Total cholesterol
**TG**	Triglyceride
**UCP1**	Uncoupling protein 1
**WAT**	White adipose tissue
**WB**	Western blot
**WE**	Wax exters
**WHO**	World health organization
**ZyE**	Zymosterol

## Data Availability

The datasets presented in this study can be found in online repositories. The names of the repository/repositories and accession number(s) can be found in the article/Supplementary Material.
