# Supplementary figures and images for: Ling-gui-zhu-gan granules reduces obesity and ameliorates metabolic disorders by inducing white adipose tissue browning in obese mice
Source: Front Physiol. 2024 Aug 2;15:1427722. doi: 10.3389/fphys.2024.1427722 (PMC11329929; doi:10.3389/fphys.2024.1427722)

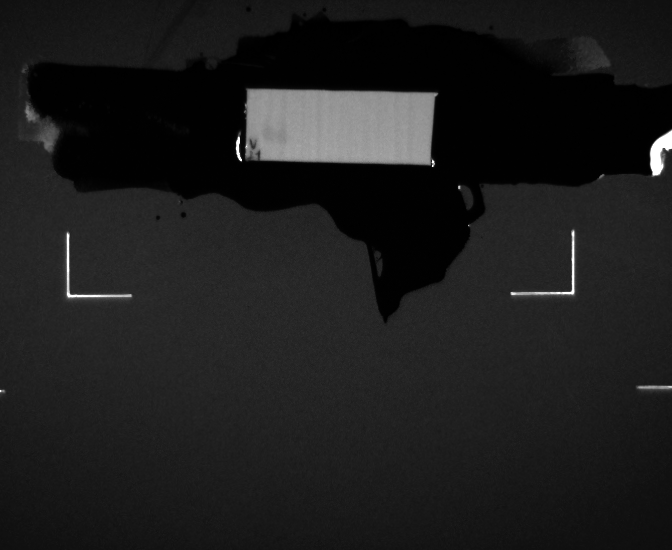

Supplement: Supplementary file 1 [file DataSheet1.ZIP › Supplementary materials/1. Original Image for Figure 4(B+C,E+F)——Western Blot/1. Figure 4(B+C)——UCP1-eWAT/Figure 4(B+C)——UCP1-1-Marker.tif]

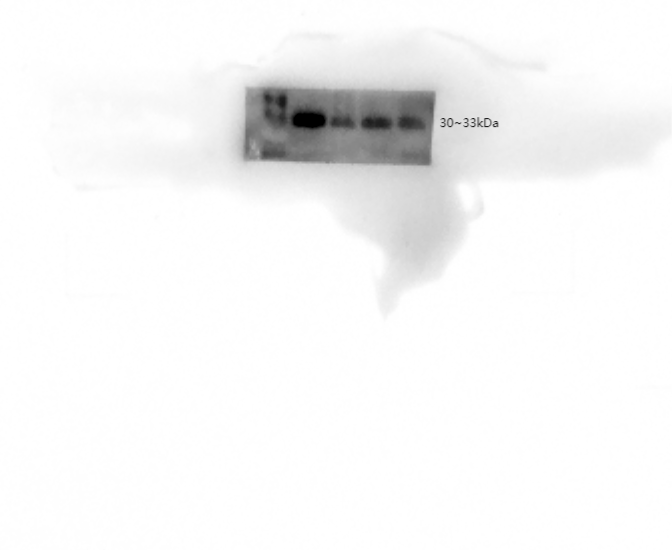

Supplement: Supplementary file 1 [file DataSheet1.ZIP › Supplementary materials/1. Original Image for Figure 4(B+C,E+F)——Western Blot/1. Figure 4(B+C)——UCP1-eWAT/Figure 4(B+C)——UCP1-1.png]

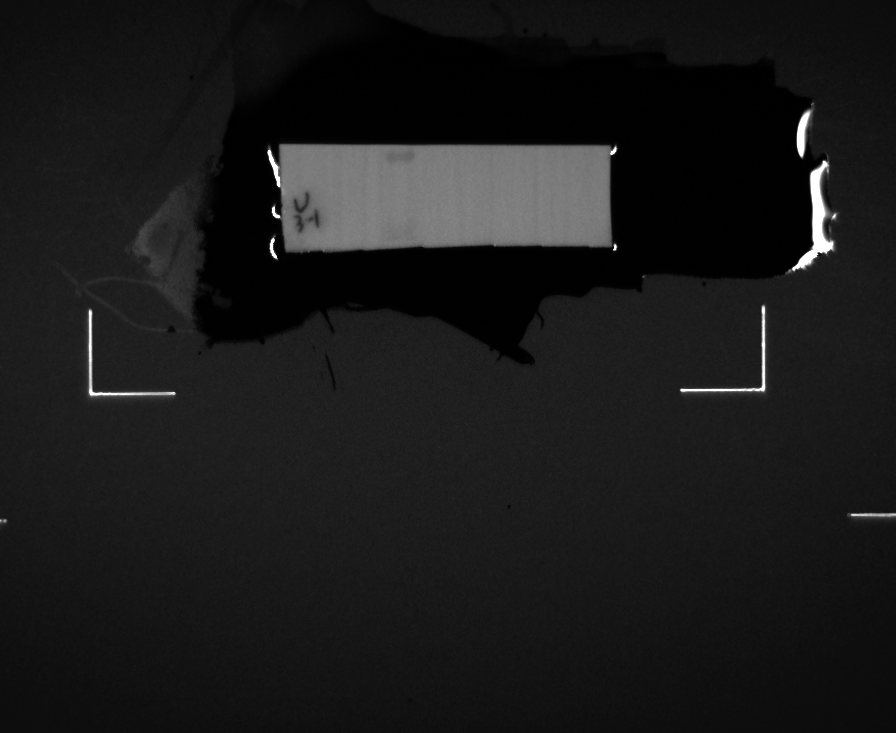

Supplement: Supplementary file 1 [file DataSheet1.ZIP › Supplementary materials/1. Original Image for Figure 4(B+C,E+F)——Western Blot/1. Figure 4(B+C)——UCP1-eWAT/Figure 4(B+C)——UCP1-2-Marker.tif]

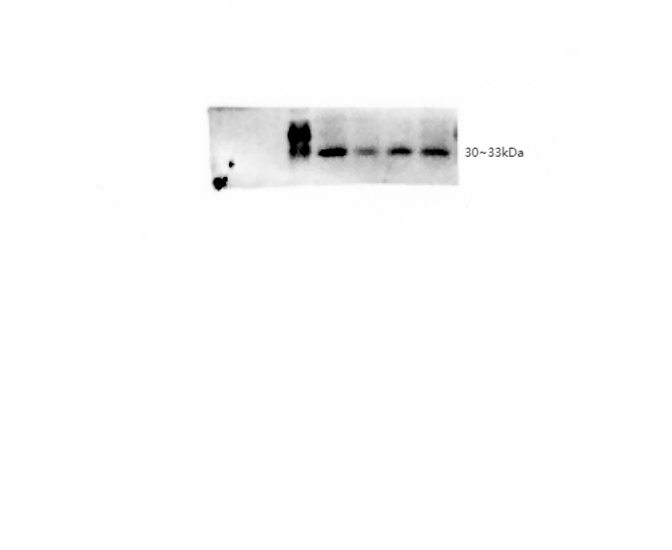

Supplement: Supplementary file 1 [file DataSheet1.ZIP › Supplementary materials/1. Original Image for Figure 4(B+C,E+F)——Western Blot/1. Figure 4(B+C)——UCP1-eWAT/Figure 4(B+C)——UCP1-2.png]

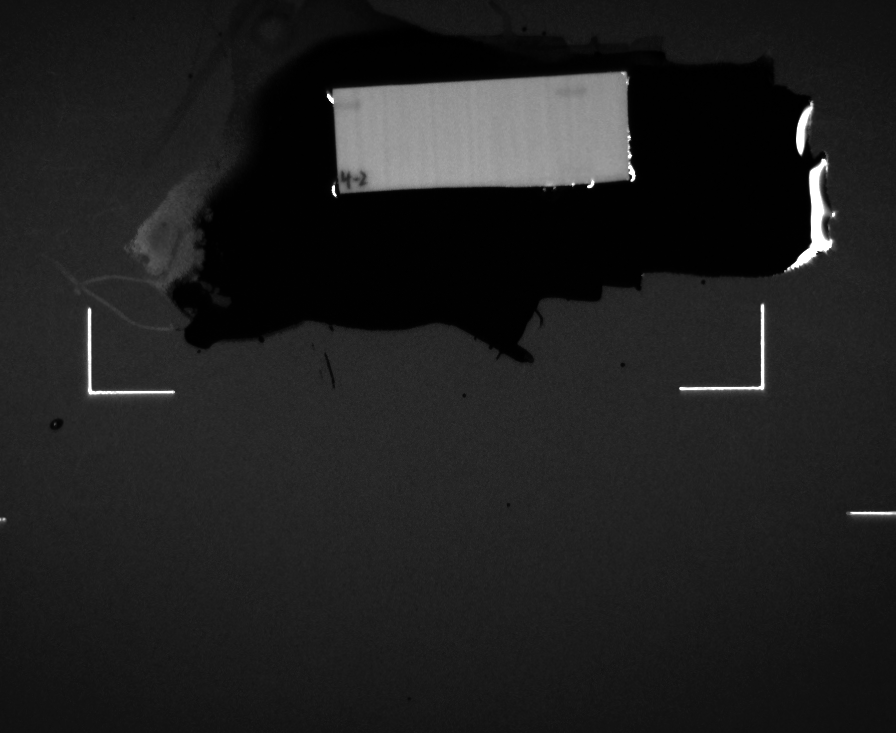

Supplement: Supplementary file 1 [file DataSheet1.ZIP › Supplementary materials/1. Original Image for Figure 4(B+C,E+F)——Western Blot/1. Figure 4(B+C)——UCP1-eWAT/Figure 4(B+C)——UCP1-3-Marker.tif]

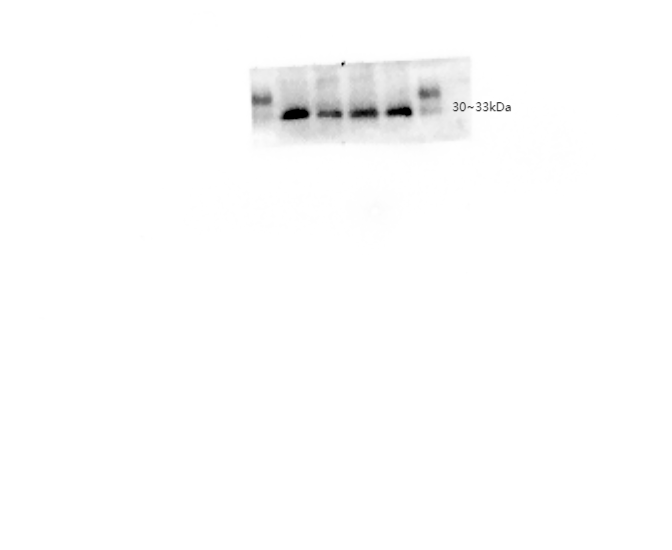

Supplement: Supplementary file 1 [file DataSheet1.ZIP › Supplementary materials/1. Original Image for Figure 4(B+C,E+F)——Western Blot/1. Figure 4(B+C)——UCP1-eWAT/Figure 4(B+C)——UCP1-3.png]

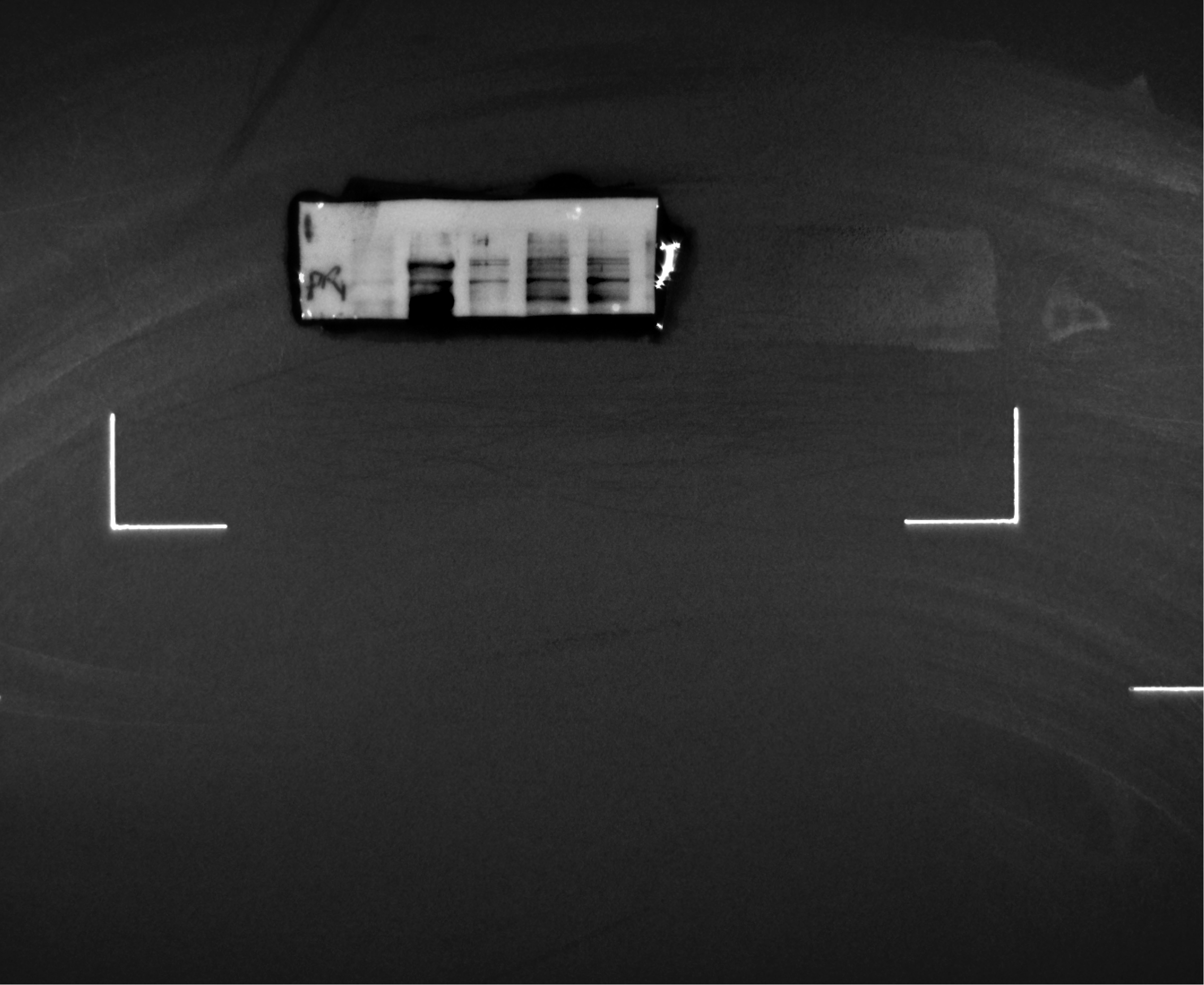

Supplement: Supplementary file 1 [file DataSheet1.ZIP › Supplementary materials/1. Original Image for Figure 4(B+C,E+F)——Western Blot/10. Figure 4(E+F)——PRDM16-sWAT/Figure 4(E+F)——PRDM16-1.tif]

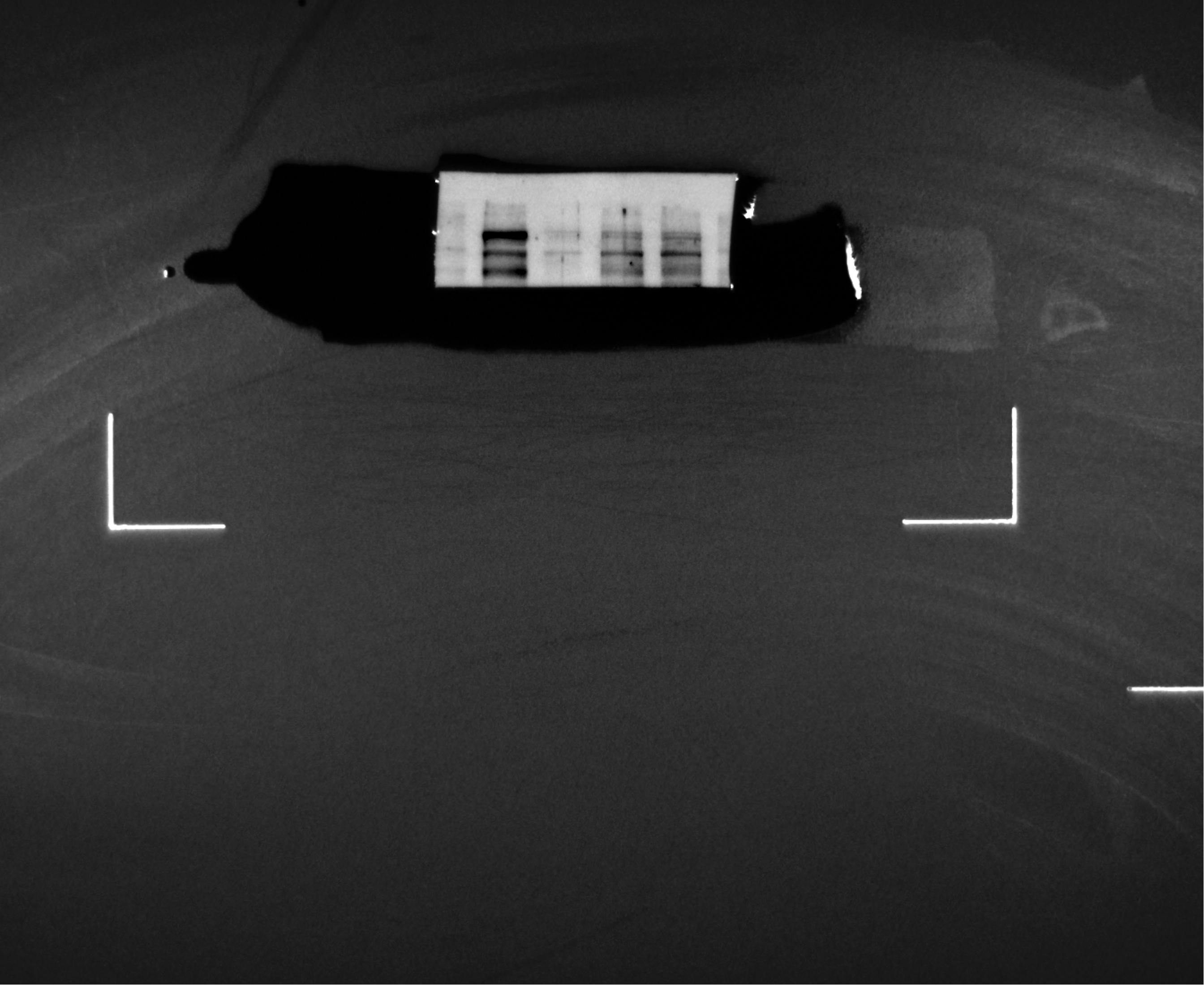

Supplement: Supplementary file 1 [file DataSheet1.ZIP › Supplementary materials/1. Original Image for Figure 4(B+C,E+F)——Western Blot/10. Figure 4(E+F)——PRDM16-sWAT/Figure 4(E+F)——PRDM16-2.tif]

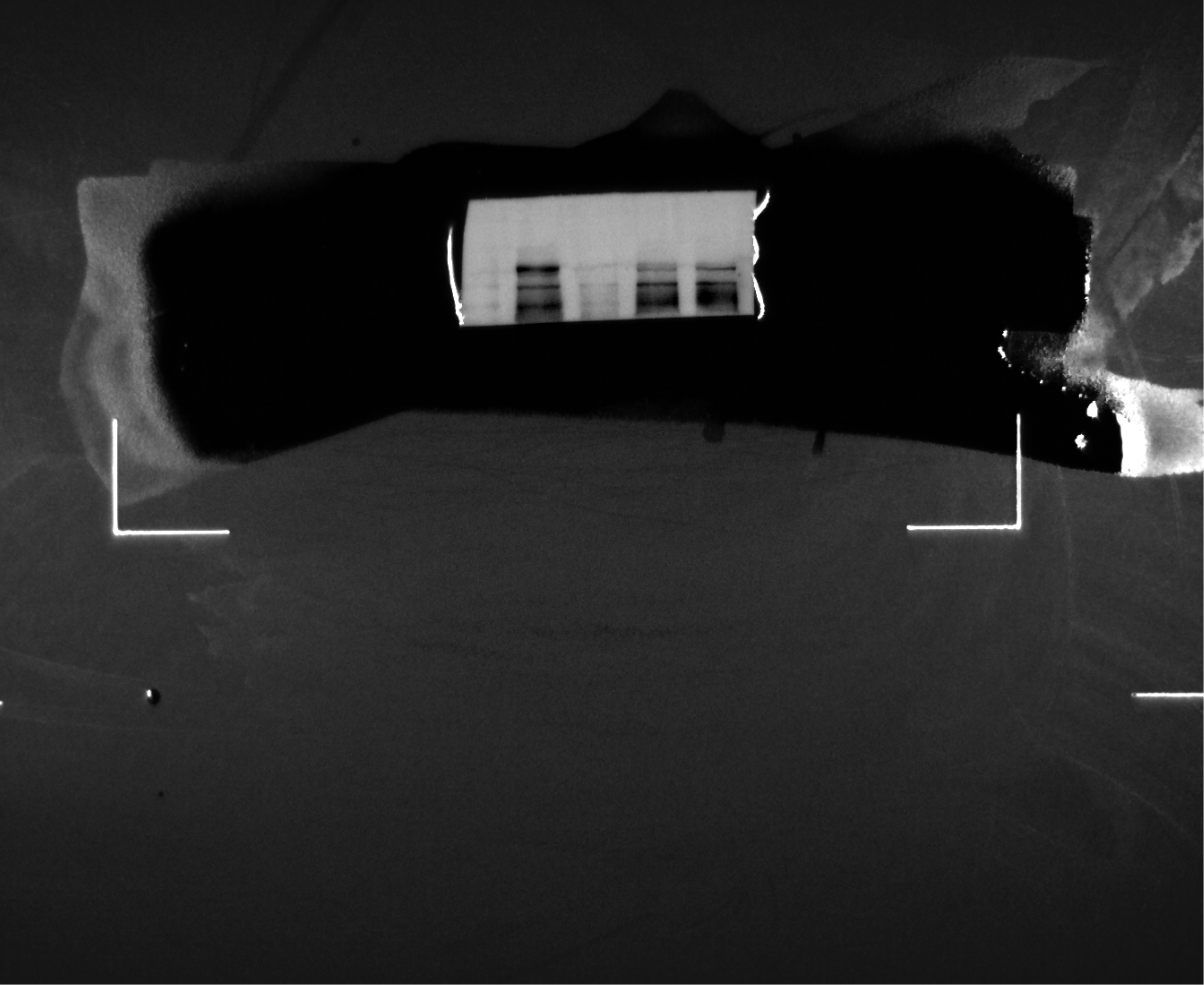

Supplement: Supplementary file 1 [file DataSheet1.ZIP › Supplementary materials/1. Original Image for Figure 4(B+C,E+F)——Western Blot/10. Figure 4(E+F)——PRDM16-sWAT/Figure 4(E+F)——PRDM16-3.tif]

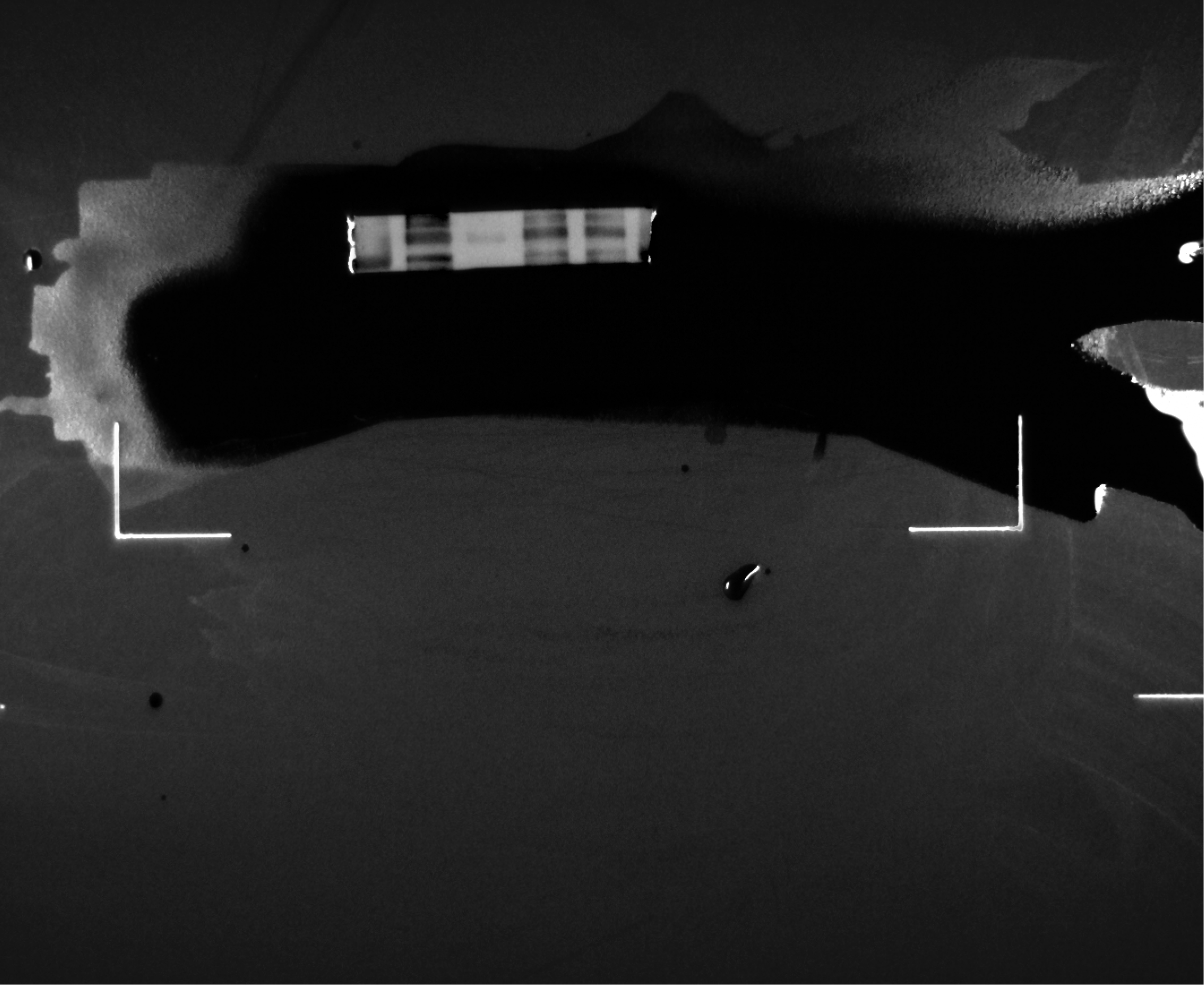

Supplement: Supplementary file 1 [file DataSheet1.ZIP › Supplementary materials/1. Original Image for Figure 4(B+C,E+F)——Western Blot/11. Figure 4(E+F)——PGC-1α-sWAT/Figure 4(E+F)——PGC-1α-1.tif]

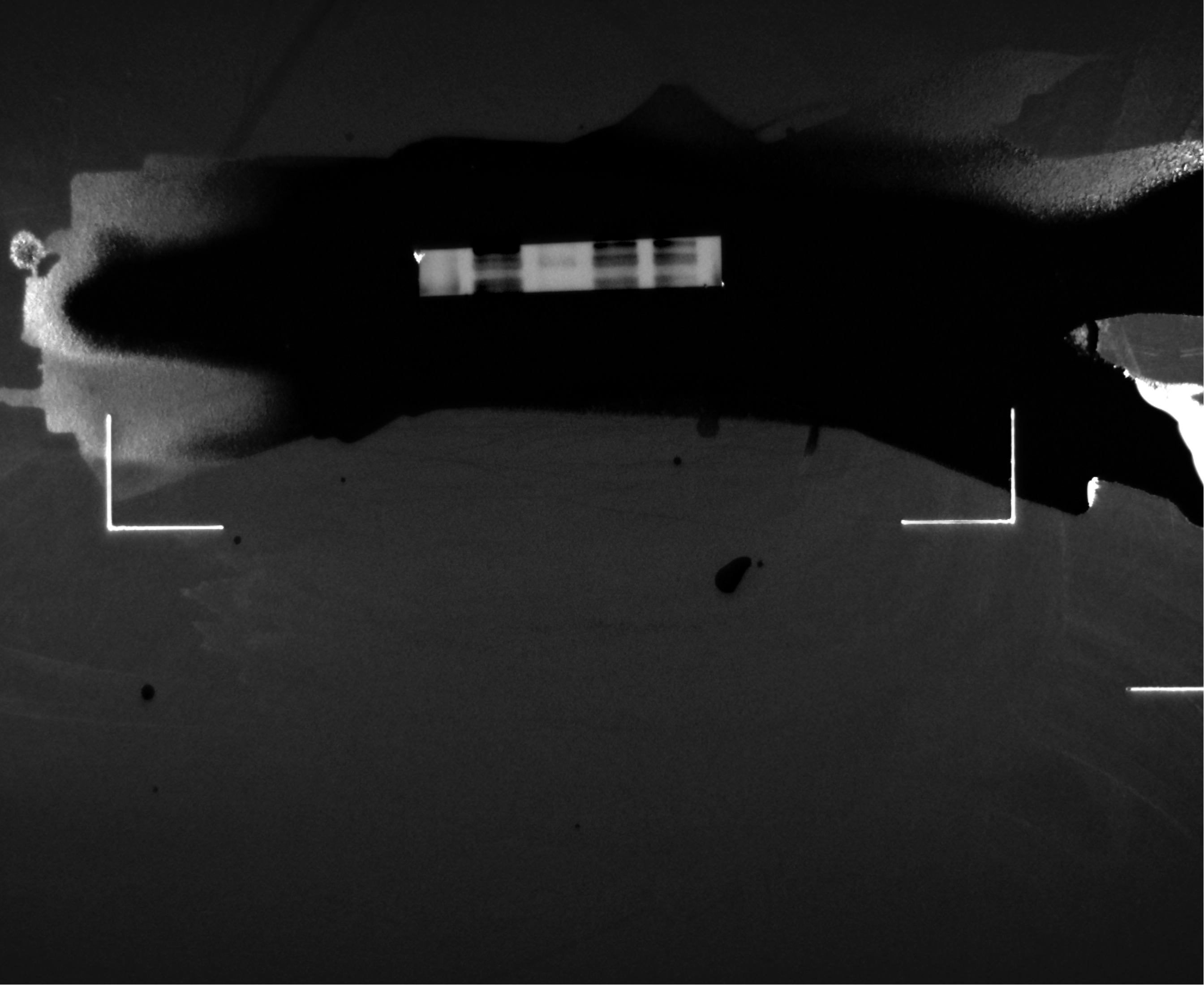

Supplement: Supplementary file 1 [file DataSheet1.ZIP › Supplementary materials/1. Original Image for Figure 4(B+C,E+F)——Western Blot/11. Figure 4(E+F)——PGC-1α-sWAT/Figure 4(E+F)——PGC-1α-2.tif]

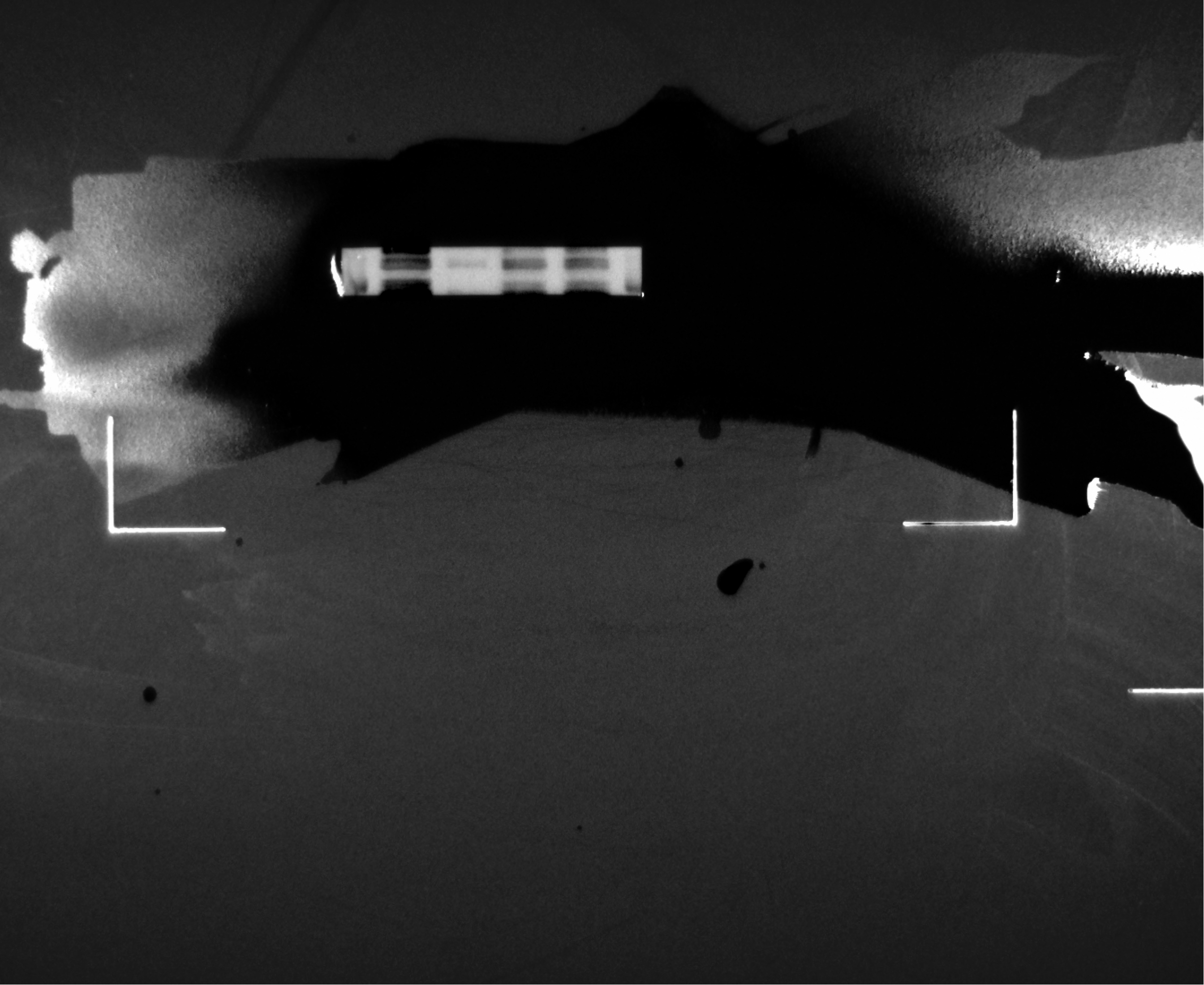

Supplement: Supplementary file 1 [file DataSheet1.ZIP › Supplementary materials/1. Original Image for Figure 4(B+C,E+F)——Western Blot/11. Figure 4(E+F)——PGC-1α-sWAT/PGC-1A-6-Marker.tif]

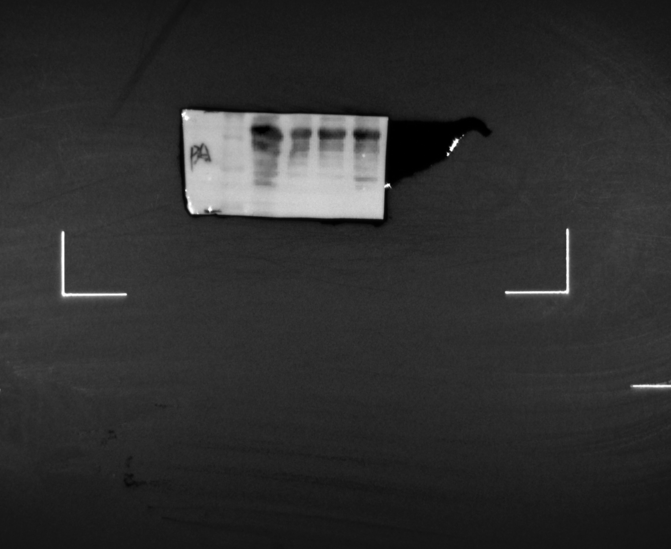

Supplement: Supplementary file 1 [file DataSheet1.ZIP › Supplementary materials/1. Original Image for Figure 4(B+C,E+F)——Western Blot/12. Figure 4(E+F)——PPARα-sWAT/Figure 4(E+F)——PPARα-1.tif]

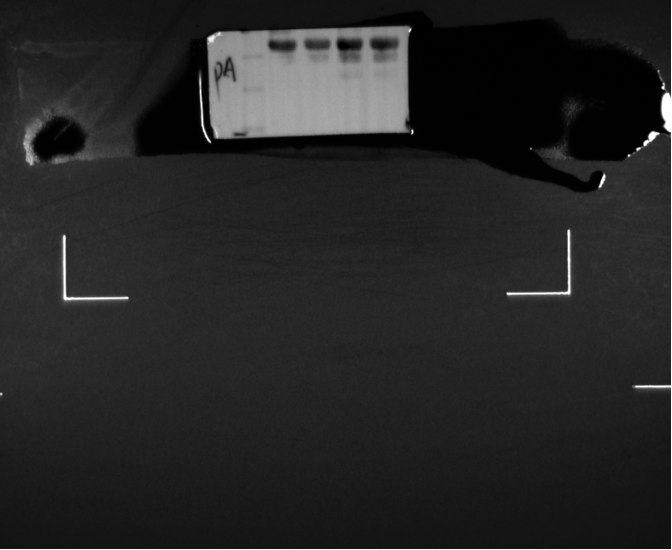

Supplement: Supplementary file 1 [file DataSheet1.ZIP › Supplementary materials/1. Original Image for Figure 4(B+C,E+F)——Western Blot/12. Figure 4(E+F)——PPARα-sWAT/Figure 4(E+F)——PPARα-2.tif]

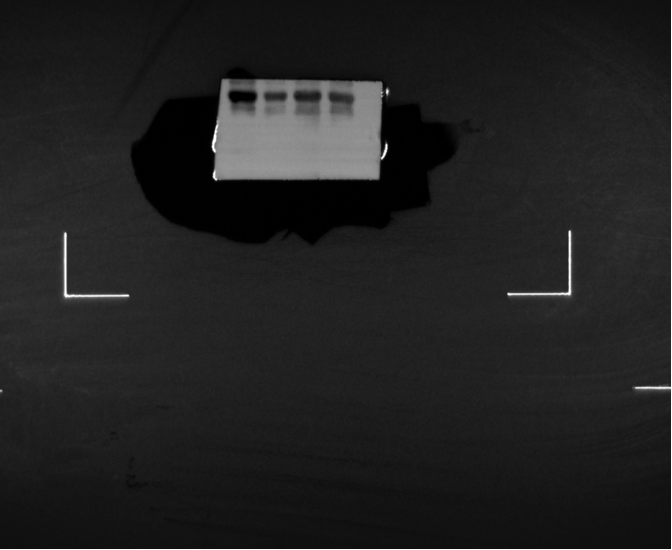

Supplement: Supplementary file 1 [file DataSheet1.ZIP › Supplementary materials/1. Original Image for Figure 4(B+C,E+F)——Western Blot/12. Figure 4(E+F)——PPARα-sWAT/Figure 4(E+F)——PPARα-3.tif]

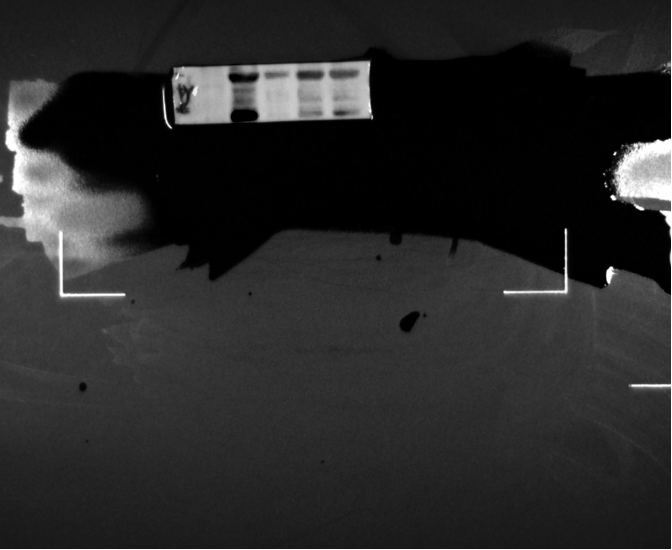

Supplement: Supplementary file 1 [file DataSheet1.ZIP › Supplementary materials/1. Original Image for Figure 4(B+C,E+F)——Western Blot/13. Figure 4(E+F)——PPARγ-sWAT/Figure 4(E+F)——PPARγ-1.tif]

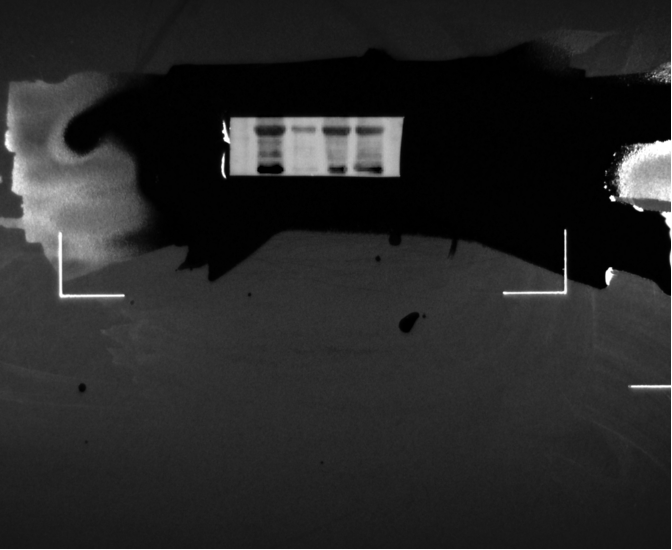

Supplement: Supplementary file 1 [file DataSheet1.ZIP › Supplementary materials/1. Original Image for Figure 4(B+C,E+F)——Western Blot/13. Figure 4(E+F)——PPARγ-sWAT/Figure 4(E+F)——PPARγ-2.tif]

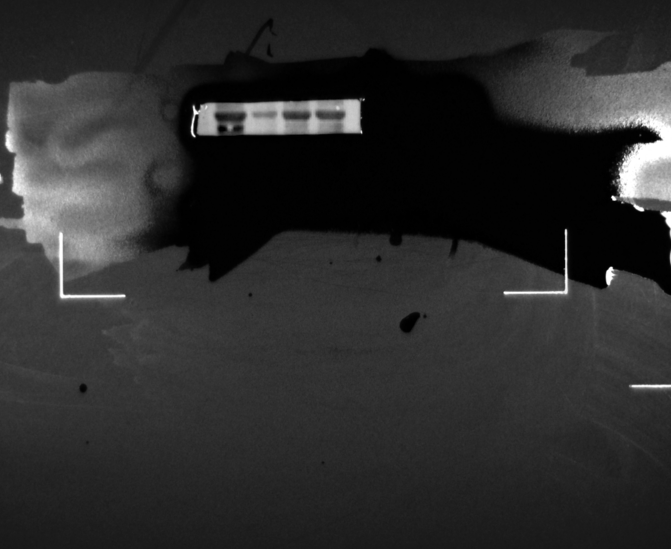

Supplement: Supplementary file 1 [file DataSheet1.ZIP › Supplementary materials/1. Original Image for Figure 4(B+C,E+F)——Western Blot/13. Figure 4(E+F)——PPARγ-sWAT/PPARy-3-Marker.tif]

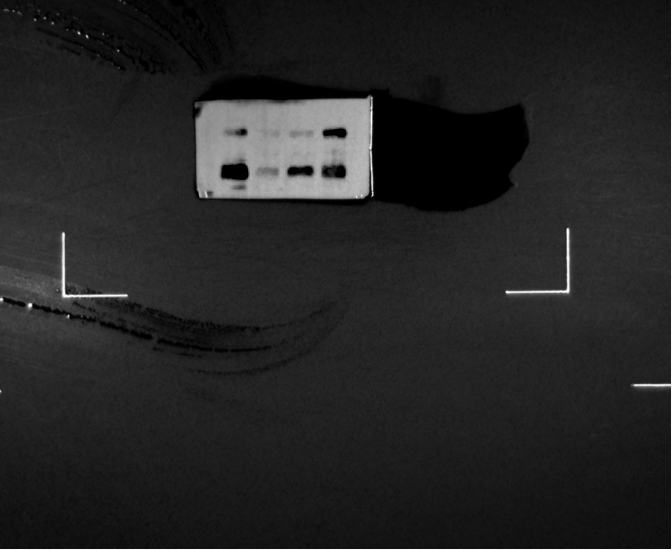

Supplement: Supplementary file 1 [file DataSheet1.ZIP › Supplementary materials/1. Original Image for Figure 4(B+C,E+F)——Western Blot/14. Figure 4(E+F)——CTBP1-sWAT/CTBP1-3-Marker.tif]

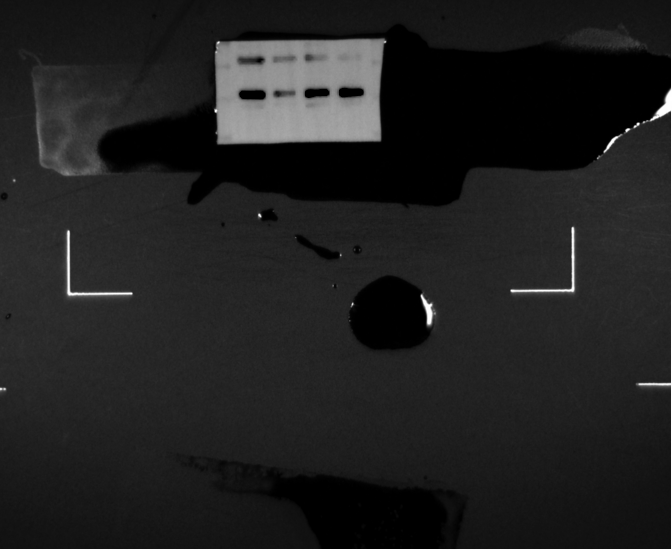

Supplement: Supplementary file 1 [file DataSheet1.ZIP › Supplementary materials/1. Original Image for Figure 4(B+C,E+F)——Western Blot/14. Figure 4(E+F)——CTBP1-sWAT/Figure 4(E+F)——CTBP1-1.tif]

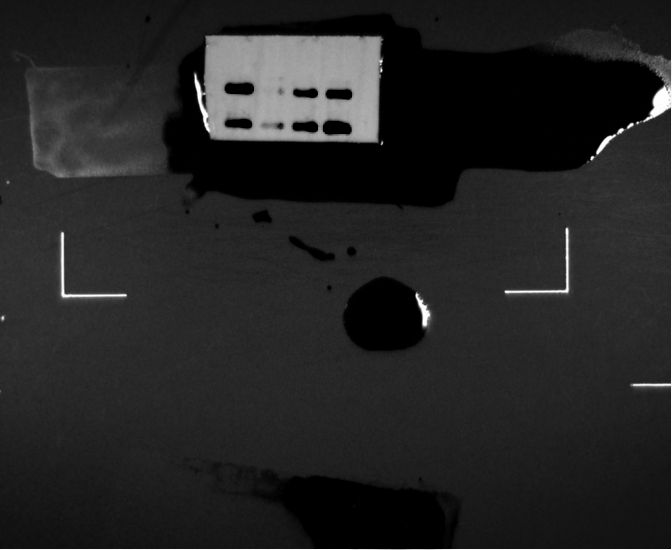

Supplement: Supplementary file 1 [file DataSheet1.ZIP › Supplementary materials/1. Original Image for Figure 4(B+C,E+F)——Western Blot/14. Figure 4(E+F)——CTBP1-sWAT/Figure 4(E+F)——CTBP1-2.tif]

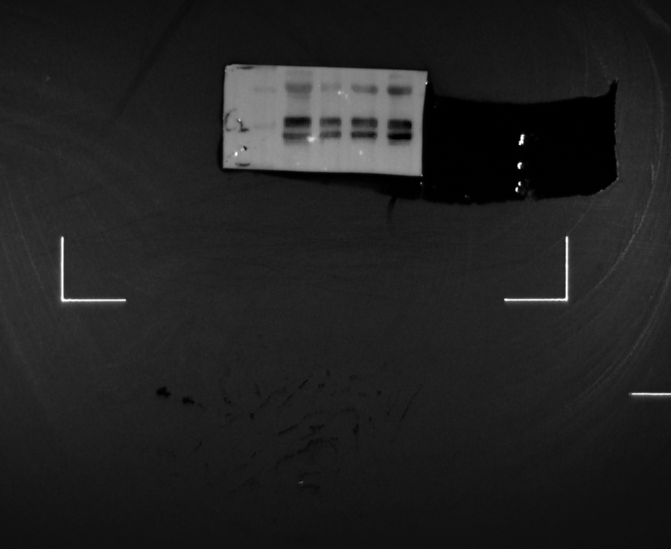

Supplement: Supplementary file 1 [file DataSheet1.ZIP › Supplementary materials/1. Original Image for Figure 4(B+C,E+F)——Western Blot/15. Figure 4(E+F)——CTBP2-sWAT/Figure 4(E+F)——CTBP2-1.tif]

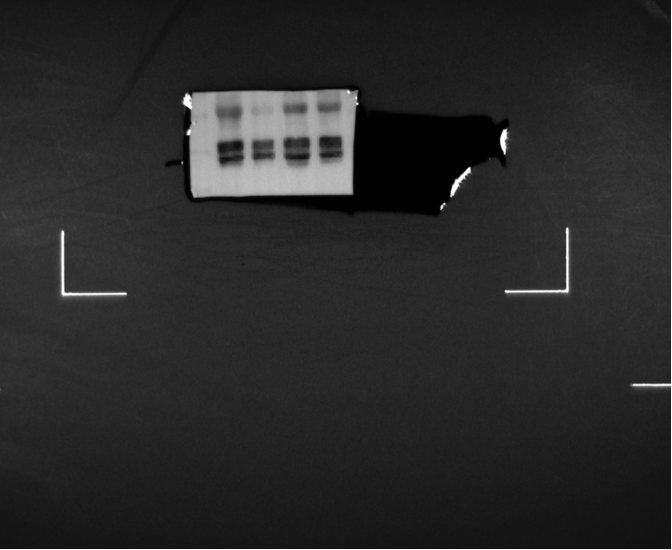

Supplement: Supplementary file 1 [file DataSheet1.ZIP › Supplementary materials/1. Original Image for Figure 4(B+C,E+F)——Western Blot/15. Figure 4(E+F)——CTBP2-sWAT/Figure 4(E+F)——CTBP2-2.tif]

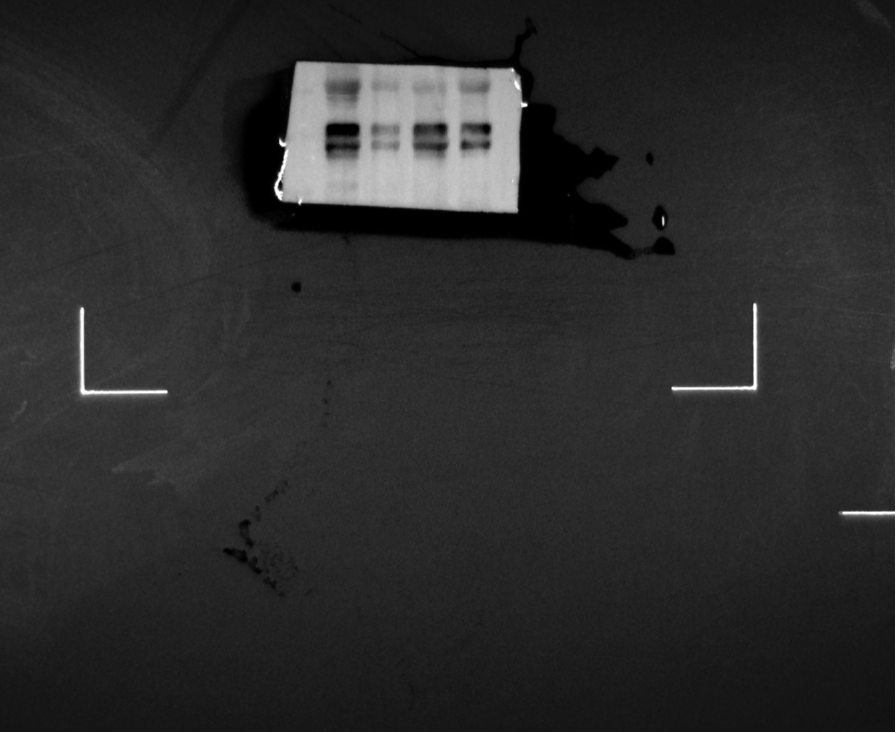

Supplement: Supplementary file 1 [file DataSheet1.ZIP › Supplementary materials/1. Original Image for Figure 4(B+C,E+F)——Western Blot/15. Figure 4(E+F)——CTBP2-sWAT/Figure 4(E+F)——CTBP2-3.tif]

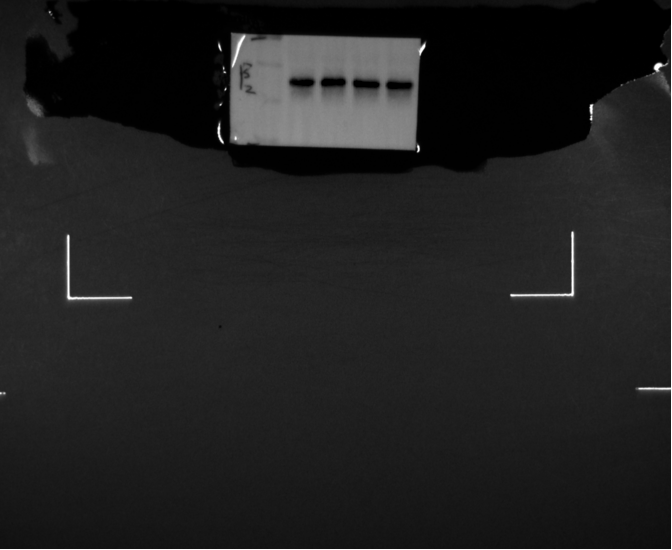

Supplement: Supplementary file 1 [file DataSheet1.ZIP › Supplementary materials/1. Original Image for Figure 4(B+C,E+F)——Western Blot/16. Figure 4(E+F)——β-Tubulin-sWAT/Figure 4(E+F)——β-Tubulin-1.tif]

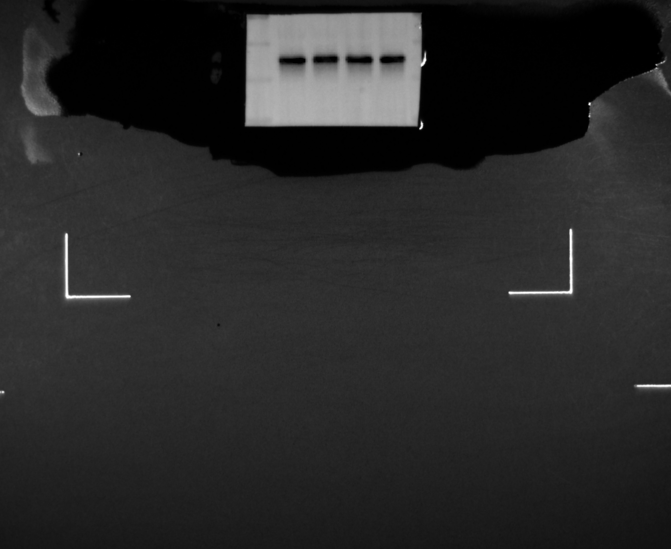

Supplement: Supplementary file 1 [file DataSheet1.ZIP › Supplementary materials/1. Original Image for Figure 4(B+C,E+F)——Western Blot/16. Figure 4(E+F)——β-Tubulin-sWAT/Figure 4(E+F)——β-Tubulin-2.tif]

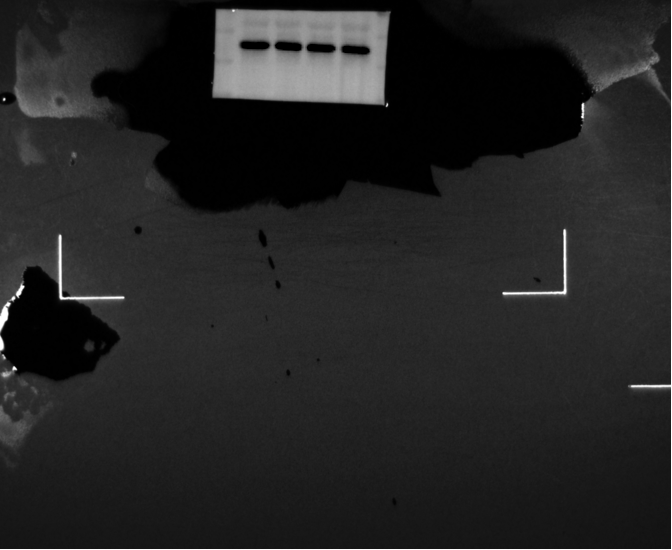

Supplement: Supplementary file 1 [file DataSheet1.ZIP › Supplementary materials/1. Original Image for Figure 4(B+C,E+F)——Western Blot/16. Figure 4(E+F)——β-Tubulin-sWAT/Figure 4(E+F)——β-Tubulin-3.tif]

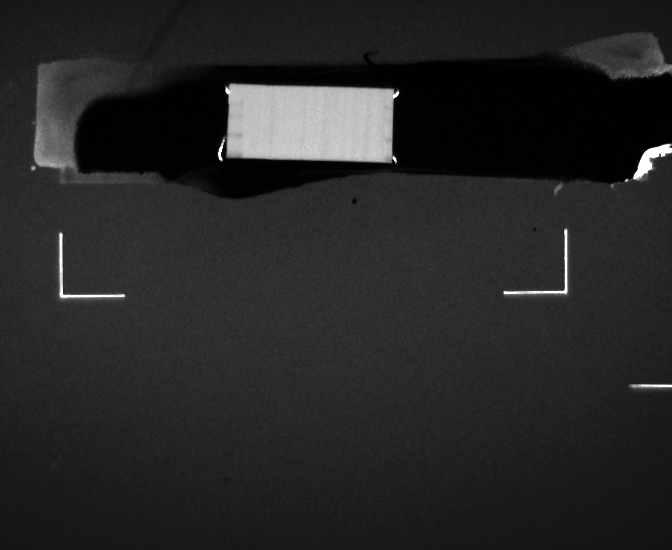

Supplement: Supplementary file 1 [file DataSheet1.ZIP › Supplementary materials/1. Original Image for Figure 4(B+C,E+F)——Western Blot/2. Figure 4(B+C)——PRDM16-eWAT/Figure 4(B+C)——PRDM16-1-Marker.tif]

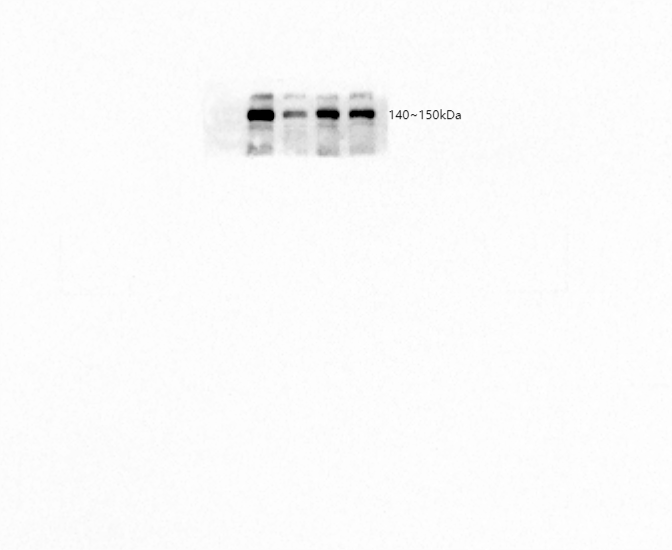

Supplement: Supplementary file 1 [file DataSheet1.ZIP › Supplementary materials/1. Original Image for Figure 4(B+C,E+F)——Western Blot/2. Figure 4(B+C)——PRDM16-eWAT/Figure 4(B+C)——PRDM16-1.png]

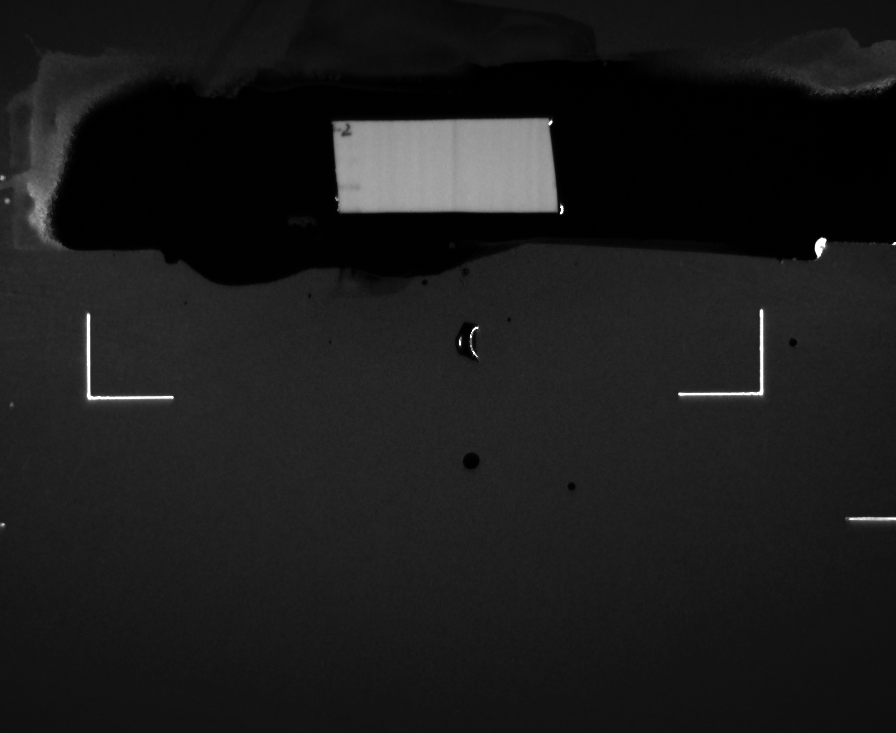

Supplement: Supplementary file 1 [file DataSheet1.ZIP › Supplementary materials/1. Original Image for Figure 4(B+C,E+F)——Western Blot/2. Figure 4(B+C)——PRDM16-eWAT/Figure 4(B+C)——PRDM16-2-Marker.tif]

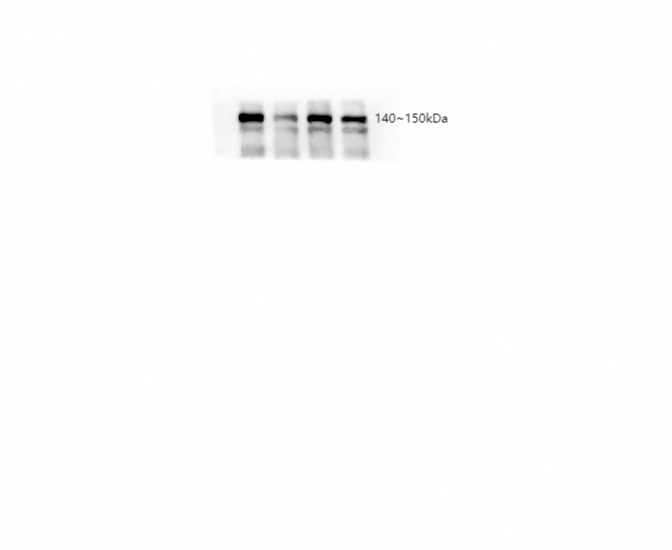

Supplement: Supplementary file 1 [file DataSheet1.ZIP › Supplementary materials/1. Original Image for Figure 4(B+C,E+F)——Western Blot/2. Figure 4(B+C)——PRDM16-eWAT/Figure 4(B+C)——PRDM16-2.png]

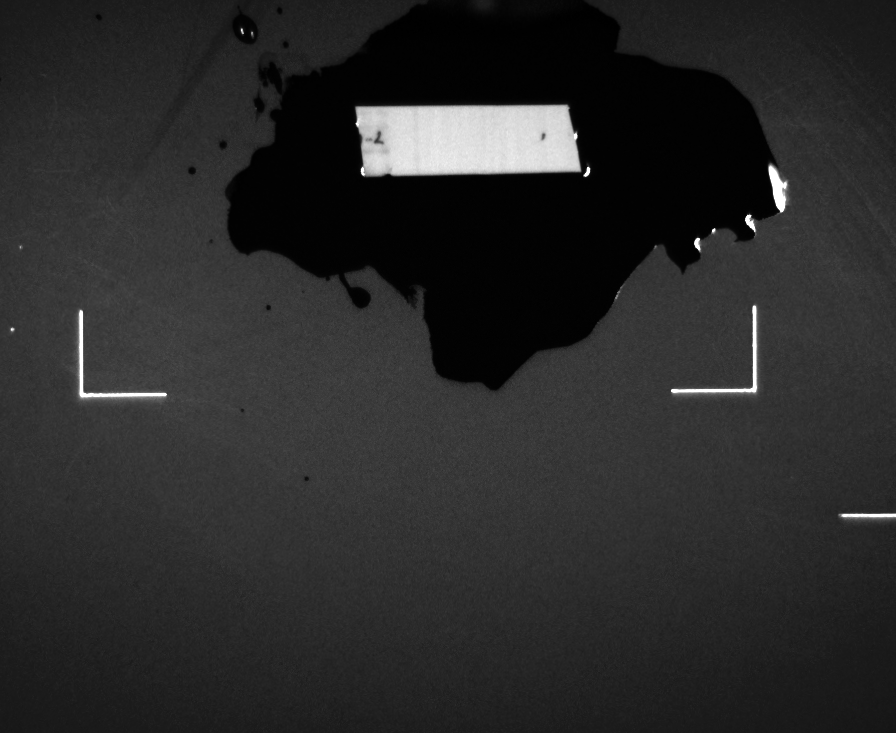

Supplement: Supplementary file 1 [file DataSheet1.ZIP › Supplementary materials/1. Original Image for Figure 4(B+C,E+F)——Western Blot/2. Figure 4(B+C)——PRDM16-eWAT/Figure 4(B+C)——PRDM16-3-Marker.tif]

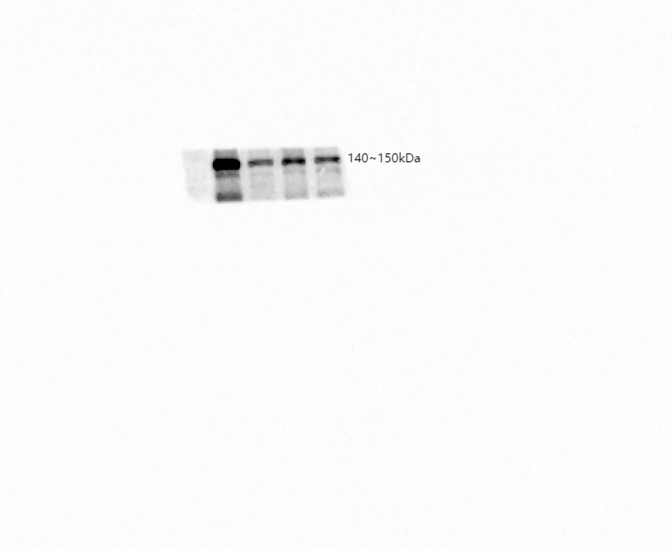

Supplement: Supplementary file 1 [file DataSheet1.ZIP › Supplementary materials/1. Original Image for Figure 4(B+C,E+F)——Western Blot/2. Figure 4(B+C)——PRDM16-eWAT/Figure 4(B+C)——PRDM16-3.png]

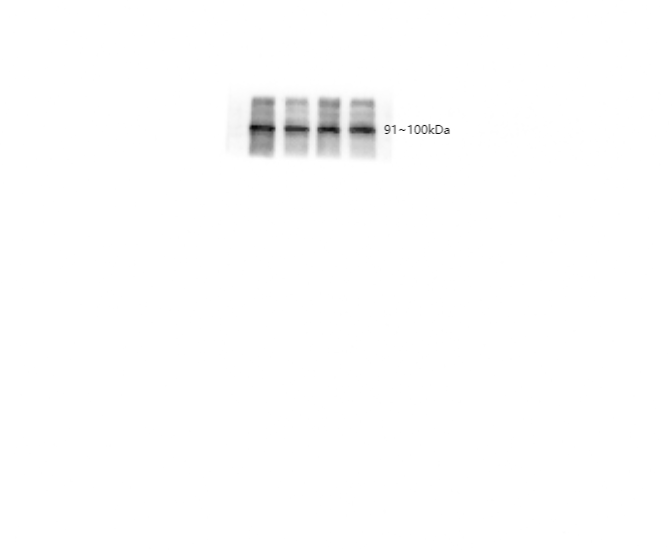

Supplement: Supplementary file 1 [file DataSheet1.ZIP › Supplementary materials/1. Original Image for Figure 4(B+C,E+F)——Western Blot/3. Figure 4(B+C)——PGC-1α-eWAT/Figure 4(B+C)——PGC-1α-1.png]

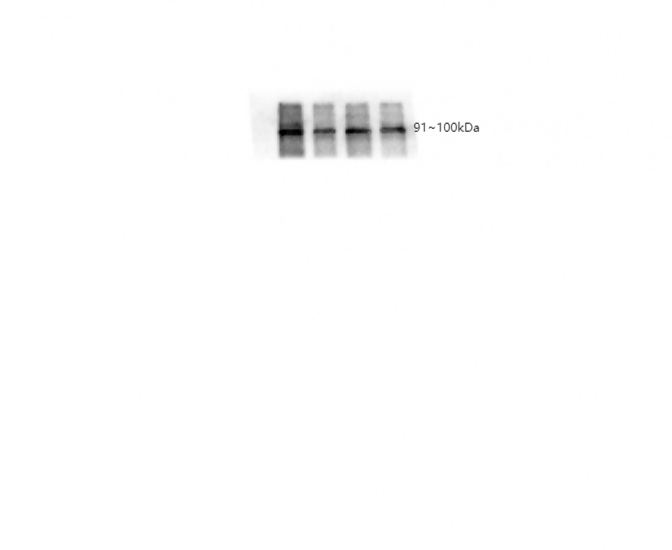

Supplement: Supplementary file 1 [file DataSheet1.ZIP › Supplementary materials/1. Original Image for Figure 4(B+C,E+F)——Western Blot/3. Figure 4(B+C)——PGC-1α-eWAT/Figure 4(B+C)——PGC-1α-2.png]

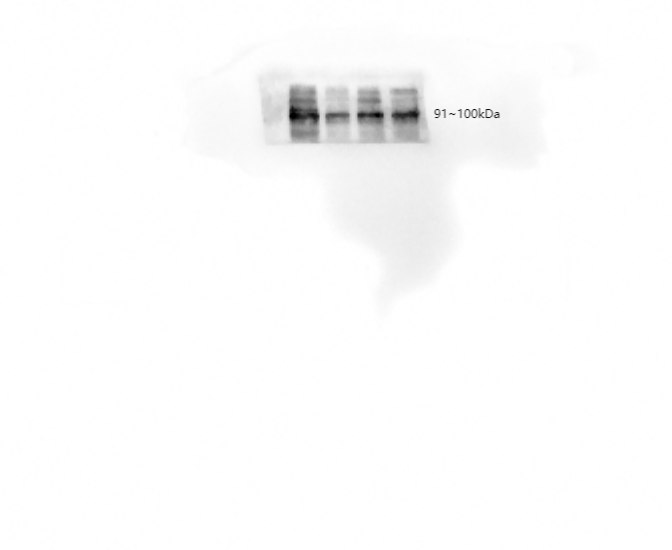

Supplement: Supplementary file 1 [file DataSheet1.ZIP › Supplementary materials/1. Original Image for Figure 4(B+C,E+F)——Western Blot/3. Figure 4(B+C)——PGC-1α-eWAT/Figure 4(B+C)——PGC-1α-3.png]

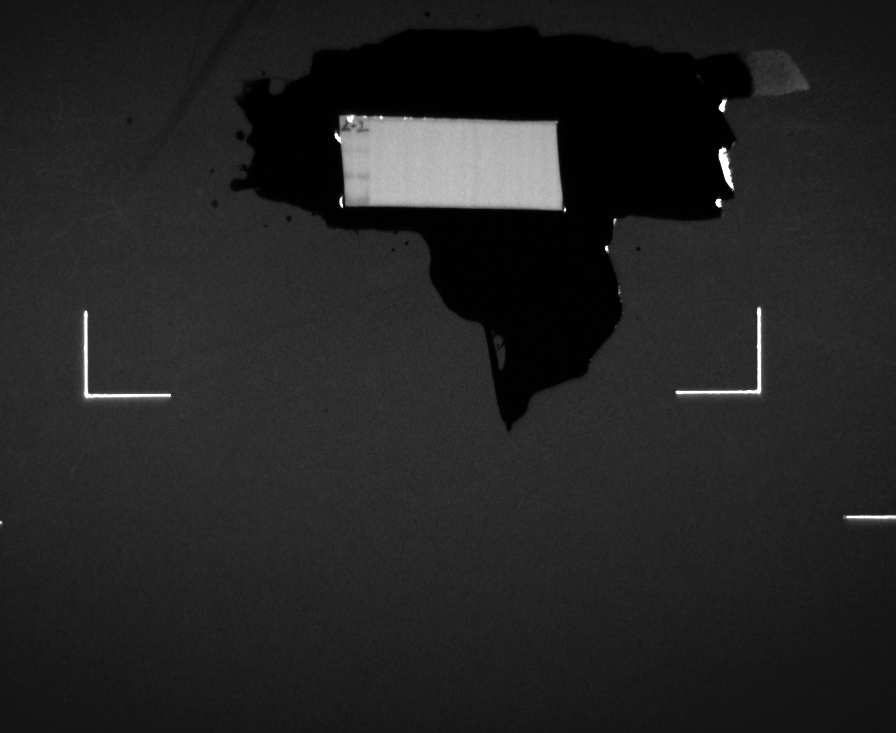

Supplement: Supplementary file 1 [file DataSheet1.ZIP › Supplementary materials/1. Original Image for Figure 4(B+C,E+F)——Western Blot/3. Figure 4(B+C)——PGC-1α-eWAT/Figure 4(J)——PGC-1α-3-Marker.tif]

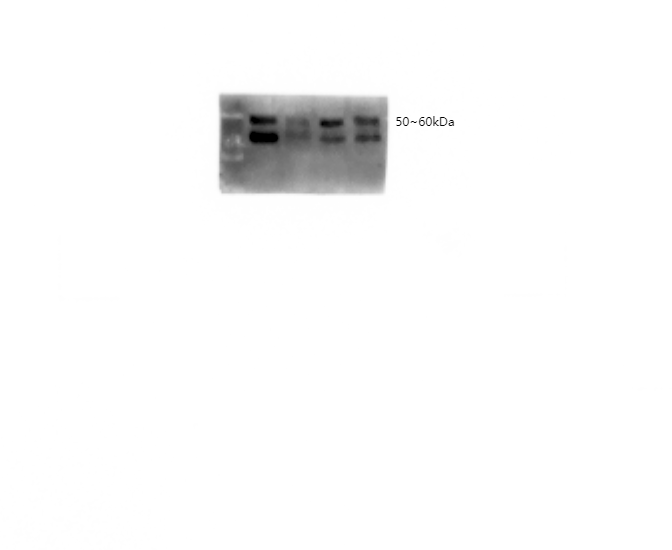

Supplement: Supplementary file 1 [file DataSheet1.ZIP › Supplementary materials/1. Original Image for Figure 4(B+C,E+F)——Western Blot/4. Figure 4(B+C)——PPARα-eWAT/Figure 4(B+C)——PPARα-1.png]

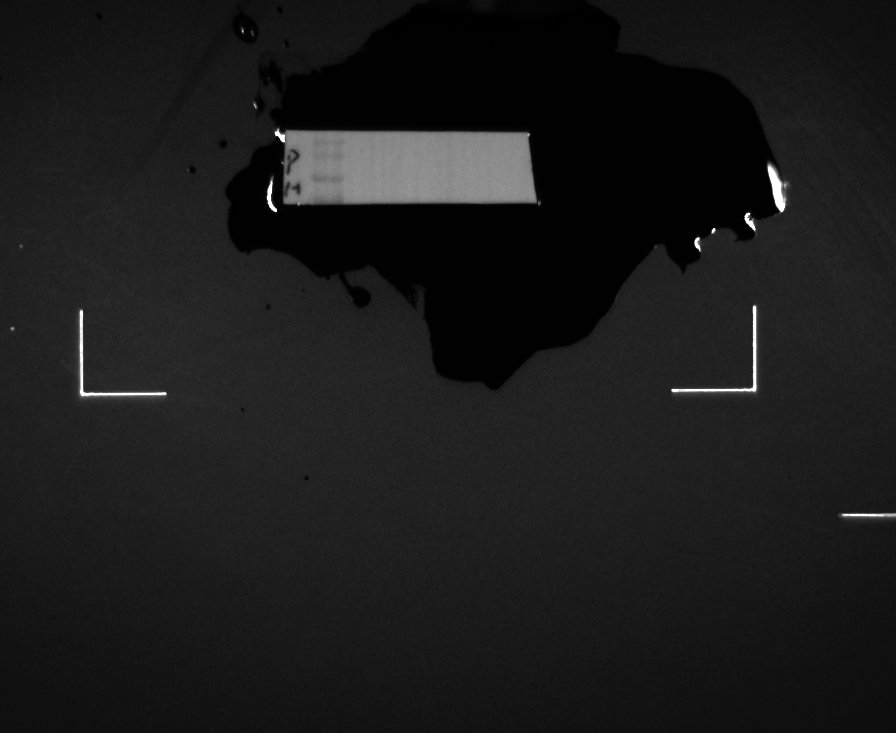

Supplement: Supplementary file 1 [file DataSheet1.ZIP › Supplementary materials/1. Original Image for Figure 4(B+C,E+F)——Western Blot/4. Figure 4(B+C)——PPARα-eWAT/Figure 4(B+C)——PPARα-2-Marker.tif]

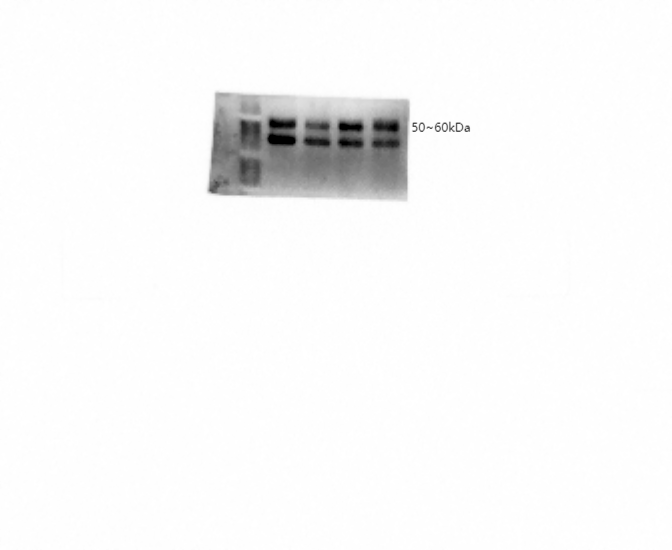

Supplement: Supplementary file 1 [file DataSheet1.ZIP › Supplementary materials/1. Original Image for Figure 4(B+C,E+F)——Western Blot/4. Figure 4(B+C)——PPARα-eWAT/Figure 4(B+C)——PPARα-2.png]

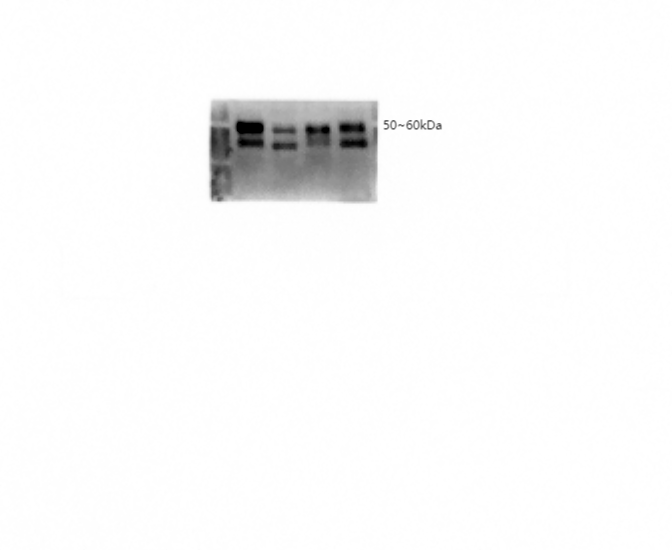

Supplement: Supplementary file 1 [file DataSheet1.ZIP › Supplementary materials/1. Original Image for Figure 4(B+C,E+F)——Western Blot/4. Figure 4(B+C)——PPARα-eWAT/Figure 4(B+C)——PPARα-3.png]

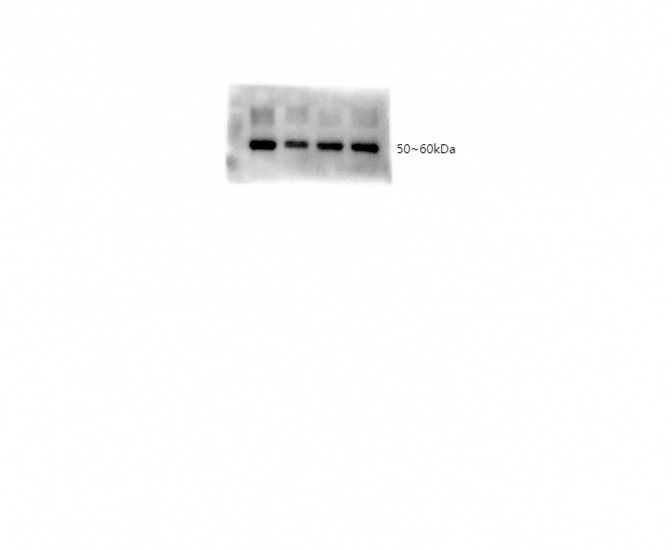

Supplement: Supplementary file 1 [file DataSheet1.ZIP › Supplementary materials/1. Original Image for Figure 4(B+C,E+F)——Western Blot/5. Figure 4(B+C)——PPARγ-eWAT/Figure 4(B+C)——PPARγ-1.png]

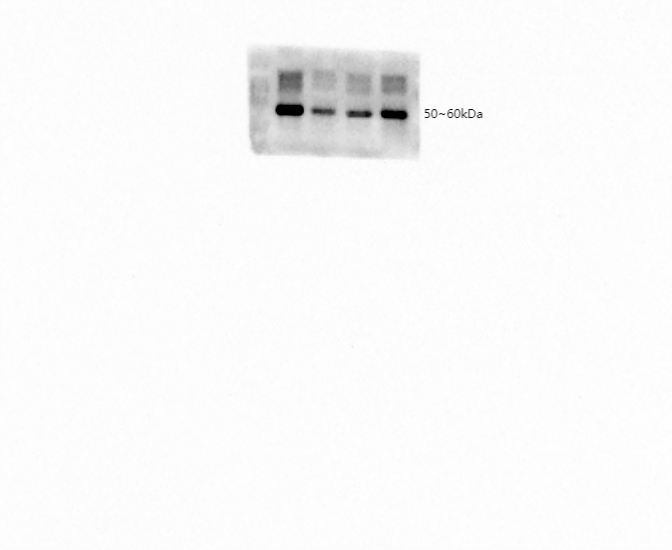

Supplement: Supplementary file 1 [file DataSheet1.ZIP › Supplementary materials/1. Original Image for Figure 4(B+C,E+F)——Western Blot/5. Figure 4(B+C)——PPARγ-eWAT/Figure 4(B+C)——PPARγ-2.png]

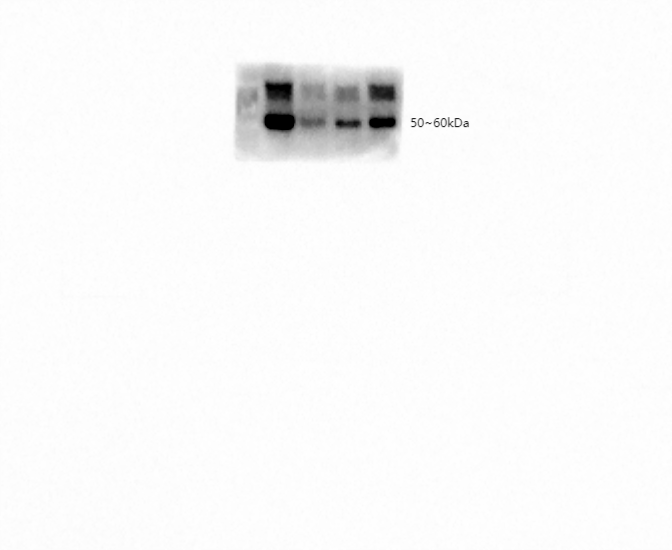

Supplement: Supplementary file 1 [file DataSheet1.ZIP › Supplementary materials/1. Original Image for Figure 4(B+C,E+F)——Western Blot/5. Figure 4(B+C)——PPARγ-eWAT/Figure 4(B+C)——PPARγ-3.png]

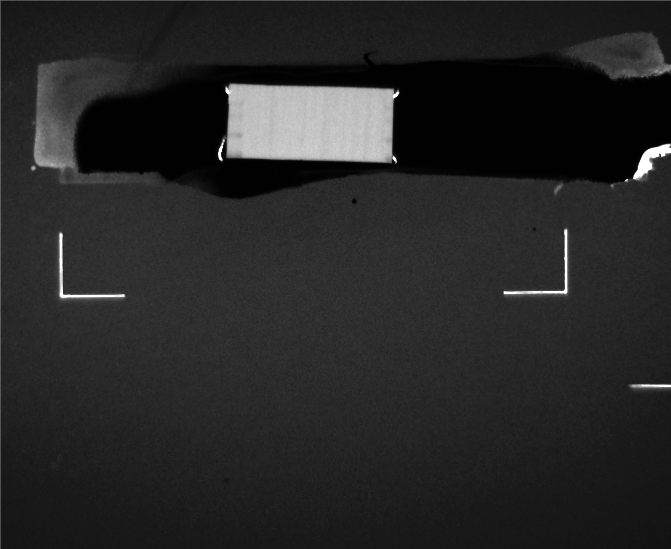

Supplement: Supplementary file 1 [file DataSheet1.ZIP › Supplementary materials/1. Original Image for Figure 4(B+C,E+F)——Western Blot/6. Figure 4(B+C)——CTBP1-eWAT/Figure 4(B+C)——CTBP1-1-Marker.png]

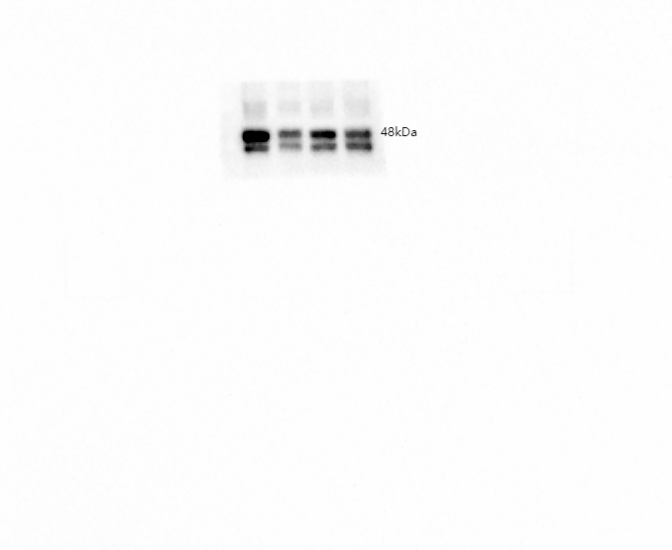

Supplement: Supplementary file 1 [file DataSheet1.ZIP › Supplementary materials/1. Original Image for Figure 4(B+C,E+F)——Western Blot/6. Figure 4(B+C)——CTBP1-eWAT/Figure 4(B+C)——CTBP1-1.png]

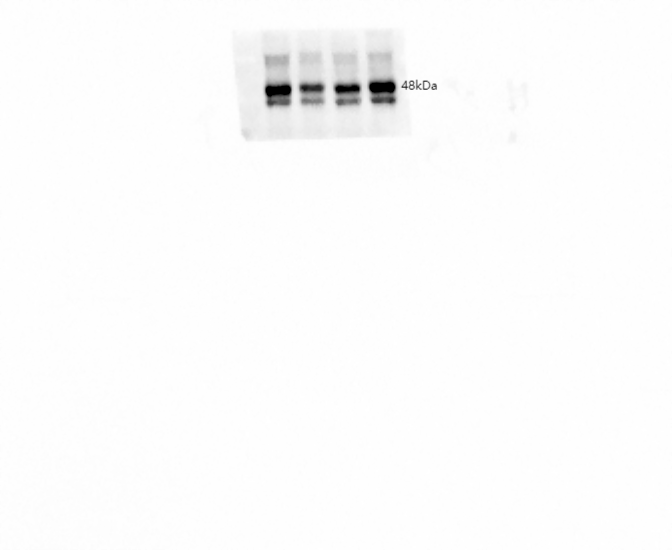

Supplement: Supplementary file 1 [file DataSheet1.ZIP › Supplementary materials/1. Original Image for Figure 4(B+C,E+F)——Western Blot/6. Figure 4(B+C)——CTBP1-eWAT/Figure 4(B+C)——CTBP1-2.png]

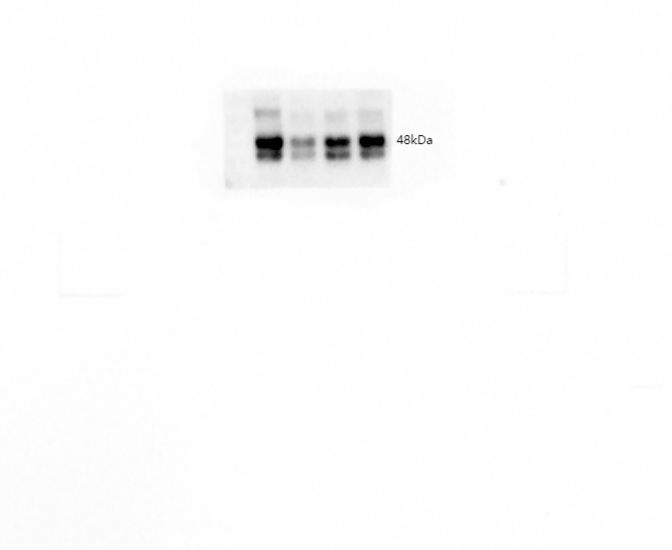

Supplement: Supplementary file 1 [file DataSheet1.ZIP › Supplementary materials/1. Original Image for Figure 4(B+C,E+F)——Western Blot/6. Figure 4(B+C)——CTBP1-eWAT/Figure 4(B+C)——CTBP1-3.png]

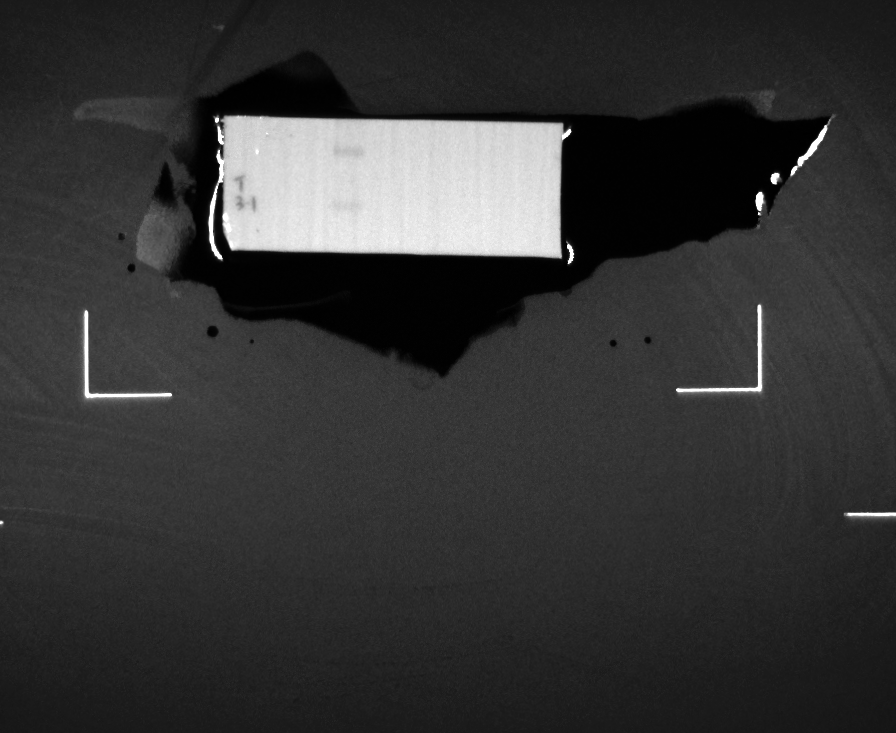

Supplement: Supplementary file 1 [file DataSheet1.ZIP › Supplementary materials/1. Original Image for Figure 4(B+C,E+F)——Western Blot/7. Figure 4(B+C)——CTBP2-eWAT/Figure 4(B+C)——CTBP2-1-Marker.tif]

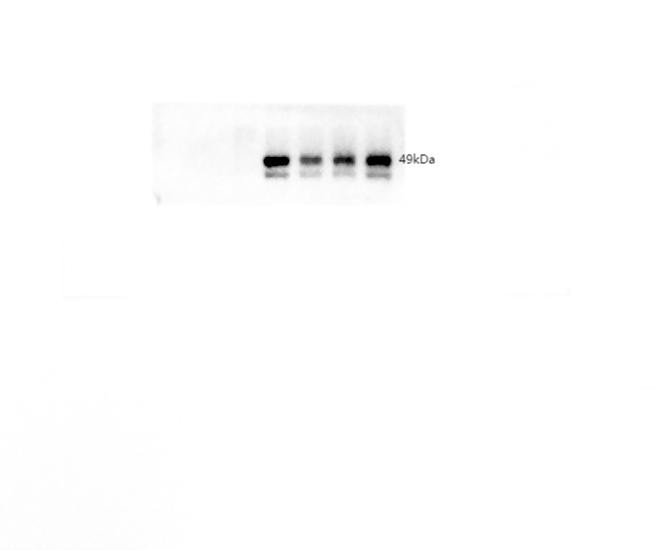

Supplement: Supplementary file 1 [file DataSheet1.ZIP › Supplementary materials/1. Original Image for Figure 4(B+C,E+F)——Western Blot/7. Figure 4(B+C)——CTBP2-eWAT/Figure 4(B+C)——CTBP2-1.png]

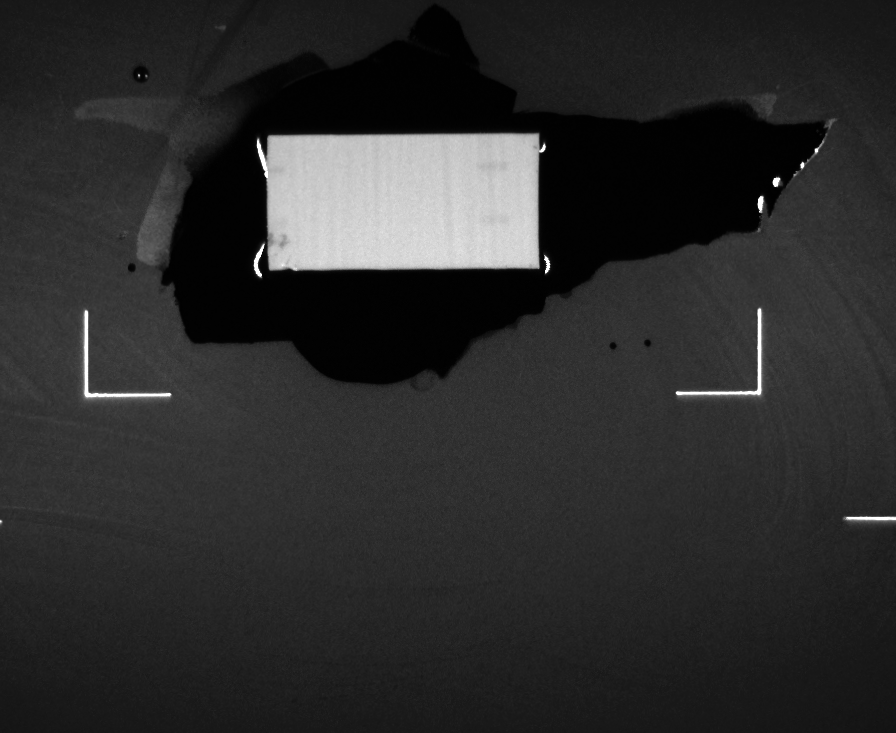

Supplement: Supplementary file 1 [file DataSheet1.ZIP › Supplementary materials/1. Original Image for Figure 4(B+C,E+F)——Western Blot/7. Figure 4(B+C)——CTBP2-eWAT/Figure 4(B+C)——CTBP2-2-Marker.tif]

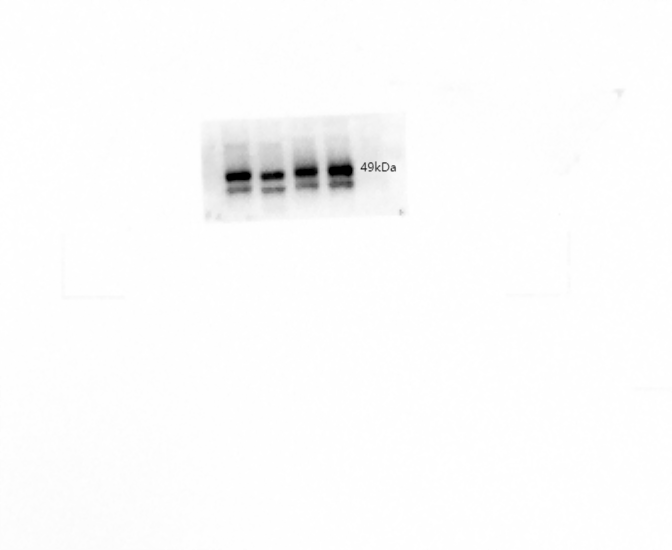

Supplement: Supplementary file 1 [file DataSheet1.ZIP › Supplementary materials/1. Original Image for Figure 4(B+C,E+F)——Western Blot/7. Figure 4(B+C)——CTBP2-eWAT/Figure 4(B+C)——CTBP2-2.png]

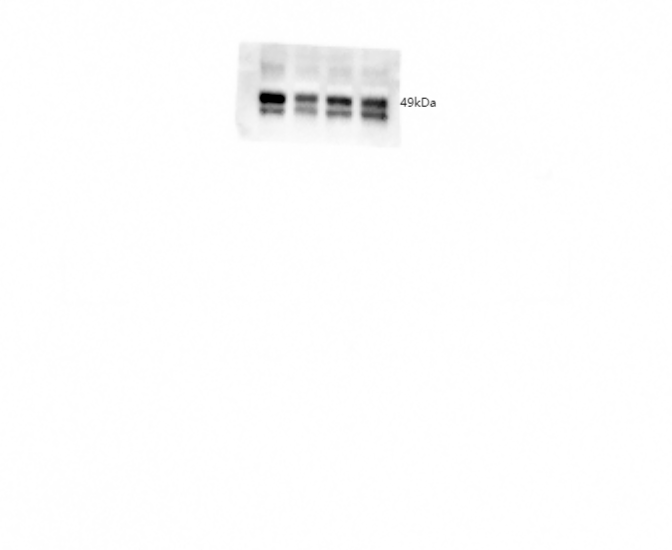

Supplement: Supplementary file 1 [file DataSheet1.ZIP › Supplementary materials/1. Original Image for Figure 4(B+C,E+F)——Western Blot/7. Figure 4(B+C)——CTBP2-eWAT/Figure 4(B+C)——CTBP2-3.png]

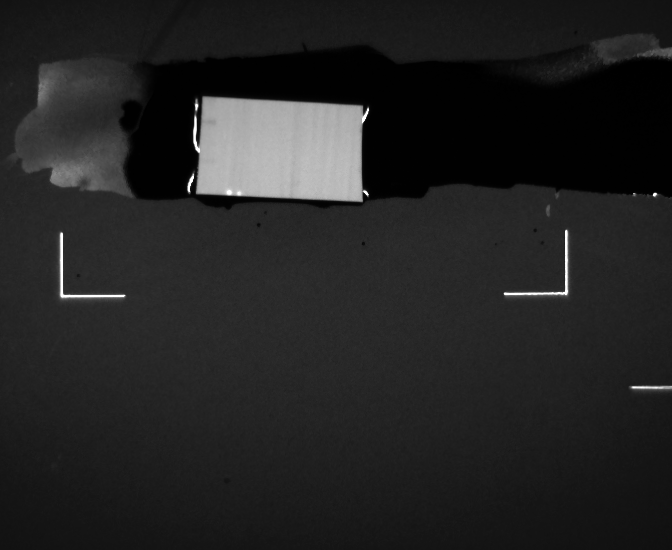

Supplement: Supplementary file 1 [file DataSheet1.ZIP › Supplementary materials/1. Original Image for Figure 4(B+C,E+F)——Western Blot/8. Figure 4(B+C)——β-Tubulin-eWAT/Figure 4(B+C)——β-Tubulin-1-Marker.tif]

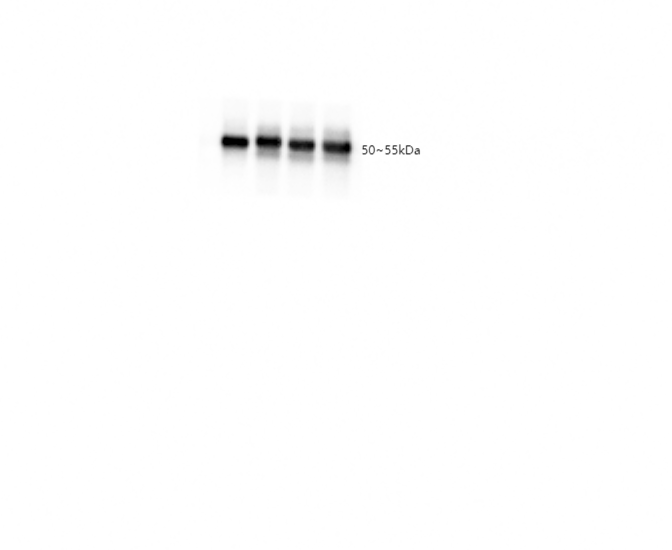

Supplement: Supplementary file 1 [file DataSheet1.ZIP › Supplementary materials/1. Original Image for Figure 4(B+C,E+F)——Western Blot/8. Figure 4(B+C)——β-Tubulin-eWAT/Figure 4(B+C)——β-Tubulin-1.png]

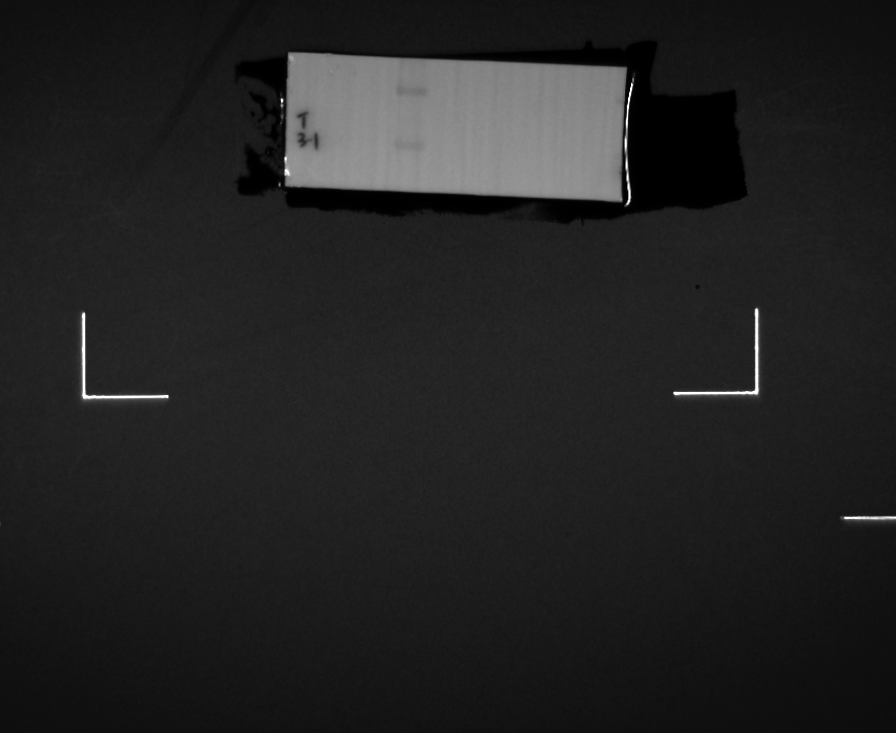

Supplement: Supplementary file 1 [file DataSheet1.ZIP › Supplementary materials/1. Original Image for Figure 4(B+C,E+F)——Western Blot/8. Figure 4(B+C)——β-Tubulin-eWAT/Figure 4(B+C)——β-Tubulin-2-Marker.tif]

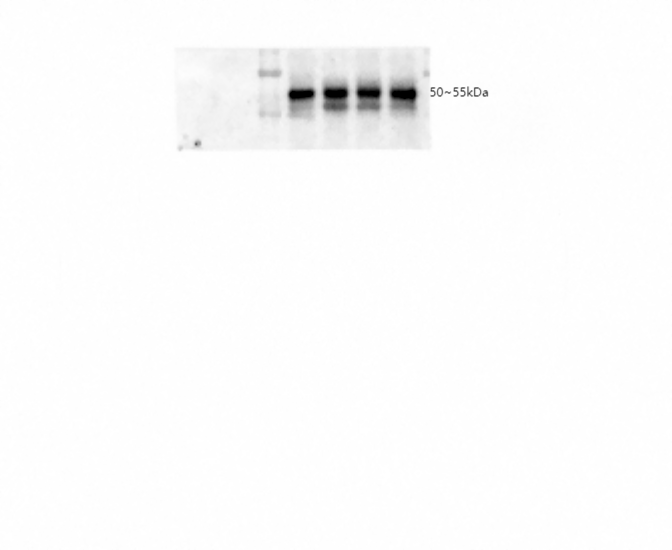

Supplement: Supplementary file 1 [file DataSheet1.ZIP › Supplementary materials/1. Original Image for Figure 4(B+C,E+F)——Western Blot/8. Figure 4(B+C)——β-Tubulin-eWAT/Figure 4(B+C)——β-Tubulin-2.png]

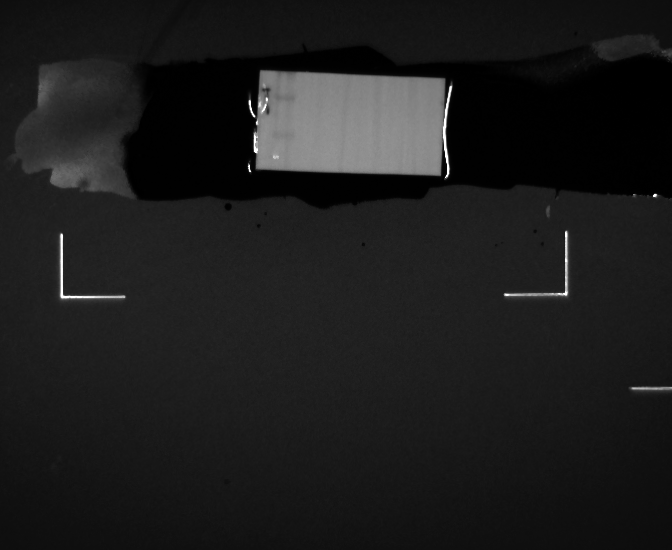

Supplement: Supplementary file 1 [file DataSheet1.ZIP › Supplementary materials/1. Original Image for Figure 4(B+C,E+F)——Western Blot/8. Figure 4(B+C)——β-Tubulin-eWAT/Figure 4(B+C)——β-Tubulin-3-Marker.tif]

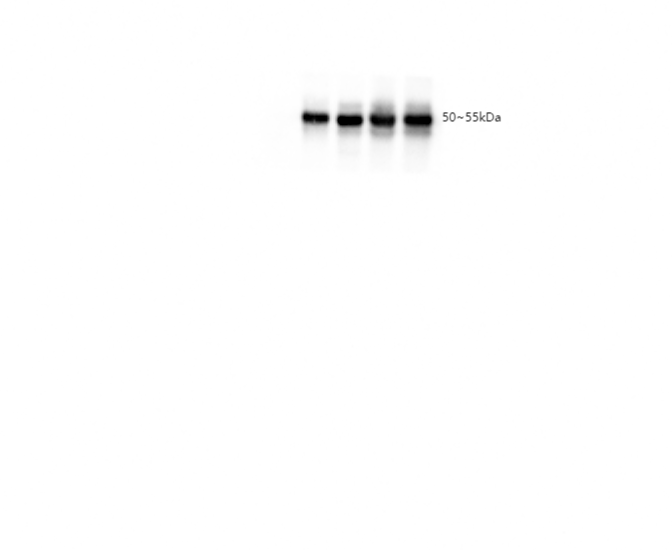

Supplement: Supplementary file 1 [file DataSheet1.ZIP › Supplementary materials/1. Original Image for Figure 4(B+C,E+F)——Western Blot/8. Figure 4(B+C)——β-Tubulin-eWAT/Figure 4(B+C)——β-Tubulin-3.png]

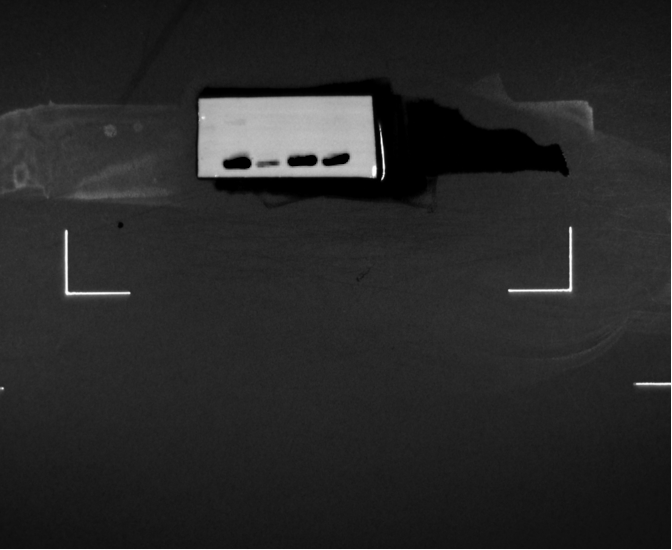

Supplement: Supplementary file 1 [file DataSheet1.ZIP › Supplementary materials/1. Original Image for Figure 4(B+C,E+F)——Western Blot/9. Figure 4(E+F)——UCP1-sWAT/Figure 4(E+F)——UCP1-1.tif]

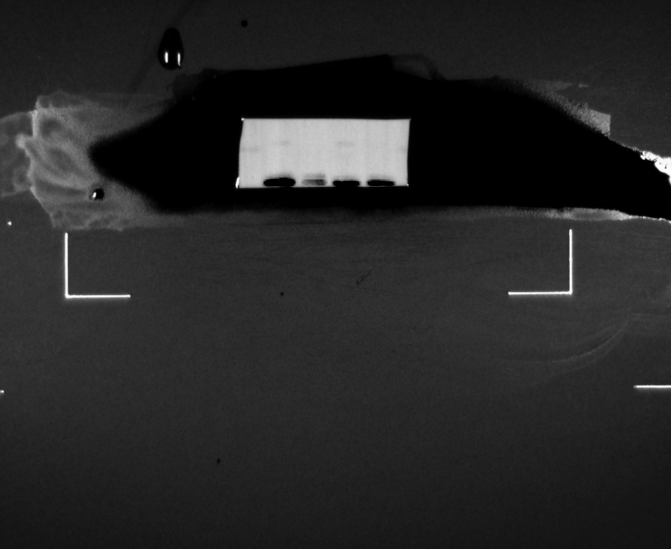

Supplement: Supplementary file 1 [file DataSheet1.ZIP › Supplementary materials/1. Original Image for Figure 4(B+C,E+F)——Western Blot/9. Figure 4(E+F)——UCP1-sWAT/Figure 4(E+F)——UCP1-2.tif]

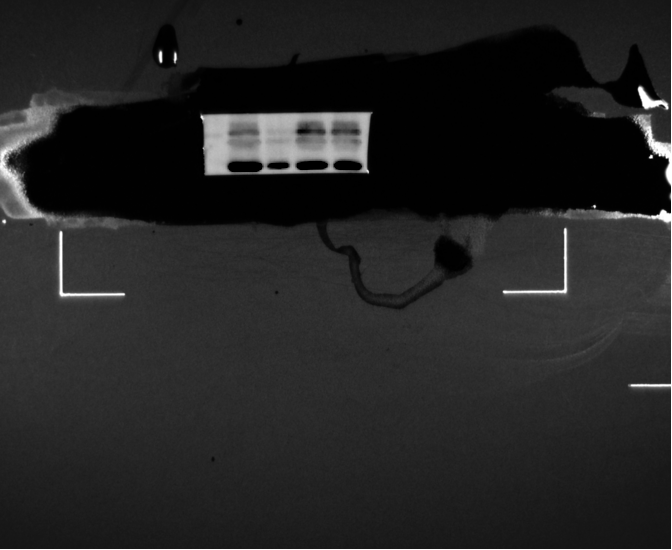

Supplement: Supplementary file 1 [file DataSheet1.ZIP › Supplementary materials/1. Original Image for Figure 4(B+C,E+F)——Western Blot/9. Figure 4(E+F)——UCP1-sWAT/Figure 4(E+F)——UCP1-3.tif]
